# Prevention strategies of postoperative adhesion in soft tissues by applying biomaterials: Based on the mechanisms of occurrence and development of adhesions

**DOI:** 10.1016/j.bioactmat.2023.02.026

**Published:** 2023-03-17

**Authors:** Jie Liao, Xiaoming Li, Yubo Fan

**Affiliations:** Key Laboratory for Biomechanics and Mechanobiology of Ministry of Education, Beijing Advanced Innovation Center for Biomedical Engineering, School of Biological Science and Medical Engineering, Beihang University, Beijing, 100083, China

**Keywords:** Prevention of postoperative adhesions (POA), Soft tissues, Mechanisms of occurrence and development, Biomaterials

## Abstract

Postoperative adhesion (POA) widely occurs in soft tissues and usually leads to chronic pain, dysfunction of adjacent organs and some acute complications, seriously reducing patients’ quality of life and even being life-threatening. Except for adhesiolysis, there are few effective methods to release existing adhesion. However, it requires a second operation and inpatient care and usually triggers recurrent adhesion in a great incidence. Hence, preventing POA formation has been regarded as the most effective clinical strategy. Biomaterials have attracted great attention in preventing POA because they can act as both barriers and drug carriers. Nevertheless, even though much reported research has been demonstrated their efficacy on POA inhibition to a certain extent, thoroughly preventing POA formation is still challenging. Meanwhile, most biomaterials for POA prevention were designed based on limited experiences, not a solid theoretical basis, showing blindness. Hence, we aimed to provide guidance for designing anti-adhesion materials applied in different soft tissues based on the mechanisms of POA occurrence and development. We first classified the postoperative adhesions into four categories according to the different components of diverse adhesion tissues, and named them as “membranous adhesion”, “vascular adhesion”, “adhesive adhesion” and “scarred adhesion”, respectively. Then, the process of the occurrence and development of POA were analyzed, and the main influencing factors in different stages were clarified. Further, we proposed seven strategies for POA prevention by using biomaterials according to these influencing factors. Meanwhile, the relevant practices were summarized according to the corresponding strategies and the future perspectives were analyzed.

## Highlights

• Classifing POA into four categories according to different components of “adhesion tissues”.
• Detailedly analyzing the process of the occurrence and development of POA in various tissues.
• Furtherly proposing seven strategies for POA prevention and summarizing relevant practices.
• Analyzing the future perspectives for POA prevention.

## Graphical abstract

**Figure:**
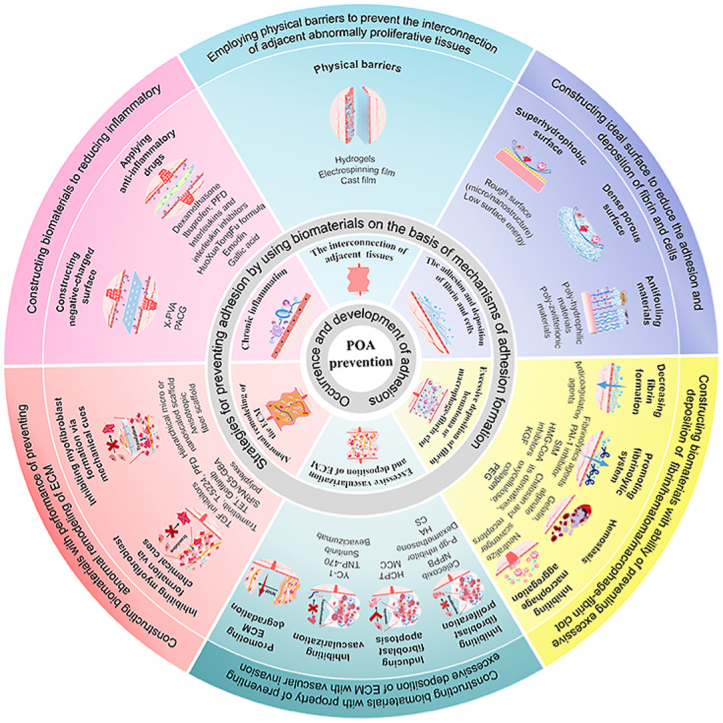

## 1 Introduction

Postoperative adhesion (POA) is various abnormal tissue hyperplasia that forms between organ and the neighboring organ or tissue following surgery trauma [1]. With the growth of adhesions, tissues would take different phenotypes ranging from a thin layer of fibrous films to a loose or dense mixture of fibrous tissues, nerves and blood vessels, and even scar tissues [2].

POA usually leads to chronic pain, dysfunction of adjacent organs (*e.g.*, intestinal obstruction, female infertility and paralysis) and some acute complications [3,4], seriously reducing life quality of patient, and even being life-threatening [[5], [6], [7]]. However, POA widely occurs in all kinds of soft tissues, especially in peritoneum [8], pericardium [9], uterine [10], dural sac [7], and tendon [11]. The corresponding pictorial diagrams, incidence conditions, incidence rates and harms were summarized in Table 1. It was reported that more than 20 million Americans needed invasive surgery every year, and around 95% of them suffered from POA [12]. Meanwhile, the adhesion-related complications led to about 1 million additional days of inpatient care annually, and over $2.5 billion had to be spent in the treatment of POA [13].

**Table 1:** The pictorial diagram, incidence condition and rate, as well as harm of common adhesion types.

Adhesion types	Pictorial Diagram	Incidence Condition	Incidence Rate	Harm
Peritoneal Adhesion	[8]	The clinical complication in abdominal and pelvic surgery [22,23].	**90%** in patients;	• Intestinal obstruction (32% of acute intestinal obstruction and 65–75% of all small bowel obstructions); • Chronic pain; • Female infertility [3,4,22,24];
			**80%**incidence of recurrent adhesion [22,23].	
Peritendinous Adhesion	[11]	The complication of tendon operations [25].	**30**–**40%** in patients [25];	• Limited movement; • Articular dyskinesia; • Perarthritis [25].
			**10%** incidence of recurrent adhesion [26].	
Cardiac Adhesion	[27]	The complication of reoperations in cardiothoracic surgery such as coronary bypass and valve repair or replacement surgeries [9].	**6**–**17%** of all coronary bypass and valve repair or replacement surgeries [28,29].	• Increasing the risk of injury to the heart, lung and great vessels; • Improving the incidence of life-threatening illnesses such as ileus and cardiac failure [26].
Epidural Adhesion	[7]	The complication of failed back surgery syndrome (FBSS) [30].	**8**–**40%** of patients under laminectomy suffer from FBSS [30]; **24%** of patients with FBSS form epidural scar [31].	• Nerve root injuries; • Leg pain or severe back pain; • Dural tears [32].
Intrauterine adhesions (IUAs)	[10,33]	The complication of the wound in the uterine cavity or the trauma to the endometrium caused by termination of pregnancy, placement of an intrauterine device (IUD), dilatation and curettage (D&C), cesarean section, or infection/irradiation [34].	**3.6%** of polyp removal, 6.7% of resection of uterine septa, 21.6% of myomectomy, 37.6% of abortion evacuation curettage [35,36];	• **Moderate or severe IUAs:** causing oligomenorrhea, amenorrhea, even infertility and severe obstetric complications including placenta previa and placenta implantation [[40], [41], [42]]; • **Asherman's syndrome (uterine cavity is completely occluded):** causing amenorrhea and infertility [43]; • Influencing women's physical and mental health.
			High rates (23% for moderate adhesion and 62% for severe adhesion) for incidence of recurrent adhesion [[37], [38], [39]].	

To release postoperative adhesion, adhesiolysis is usually applied clinically. However, a second operation and inpatient care would be required, which could affect life quality and increase medical costs [14]. It was reported that the United States paid over $2.1 billion annually in adhesiolysis, with 100 additional days of hospital readmission every year [15,16]. Moreover, recurrent adhesion, which was reported to be more complicated and difficult to prevent than primary adhesion, would be triggered in a great incidence of at least 80% after adhesiolysis surgery because of the new trauma generated during surgical lysis [17]. Until now, clinical treatments are focused on relieving the primary adhesion, and few of them performed enough efficacy in alleviating the recurrent adhesion after adhesiolysis [18,19].

Hence, effective post-operative adhesion prevention is the most effective clinical strategy and is critical to improving surgical outcomes, reducing patient pain and reoperation rates, and saving prognostic costs, which has been a major unmet clinical requirement.

At present, barrier systems or pharmacological treatments are the two main strategies for preventing postoperative adhesions [20]. Since biomaterials can act as both barriers and drug carriers, they have attracted great attentions in preventing POA in the past 30 years. Sanjoy et al. [21] studied the economic impact of the usage of the GYNECARE INTERCEED® absorbable adhesion barrier in preventing adhesions following open gynecologic surgeries (*e.g.* Caesarean section surgery, endometriosis surgery, ovarian surgery, tubal surgery, myomectomy, and hysterectomy), and found that a net savings of $540,823 could be estimated if one GYNECARE INTERCEED absorbable adhesion barrier sheet was applied per surgery in 600 gynecologic surgeries in a 3-year period, resulting in significant savings for hospitals.

Various of biomaterials have been designed and prepared as anti-adhesion barriers, which are aimed to prevent POA from the following four aspects: (1) separating adjacent injured tissues or organs (as physical barrier); (2) inhibiting fibroblast proliferation and collagen synthesis; (3) preventing protein adhesions; (4) reducing the inflammation [44]. However, because of the complexity of the causes of POA and the diversity of the tissues, anti-adhesive biomaterials prepared under the guidance of simple anti-adhesion mechanisms are difficult to meet diverse clinical needs. Currently, several reviews have paid attention on adhesion prevention, while some of them only summarized the antiadhesive materials from the perspective of composition and construction, lacking the analysis on the mechanism of antiadhesion effectiveness [9,34,45]; The other devoted to discussing anti-adhesion film for one specific tissue, and couldn't meet the requirements of adhesion prevention in other tissues [4,23,46]; And others analyzed the mechanisms of POA formation and summarized the relevant practices, while the relationship between the mechanisms and practices was not obvious [20]. Therefore, it is necessary to summarize the universal causes, processes, outcomes and influencing factors of soft tissues postoperative adhesion to provide a stronger basis for the design and preparation of anti-adhesive barriers and to achieve desired anti-adhesive effect.

This review is aimed to summarize and analyze the universal mechanisms of various types of tissue adhesions. Further, possible anti-adhesion strategies through applicating biomaterials were proposed. Meanwhile, the relevant practices according to the corresponding strategies and their anti-adhesion effects were summarized. And, the future perspectives were analyzed.

## 2 Occurrence and development of adhesions from a pathophysiological point of view

**Fig. 1:**
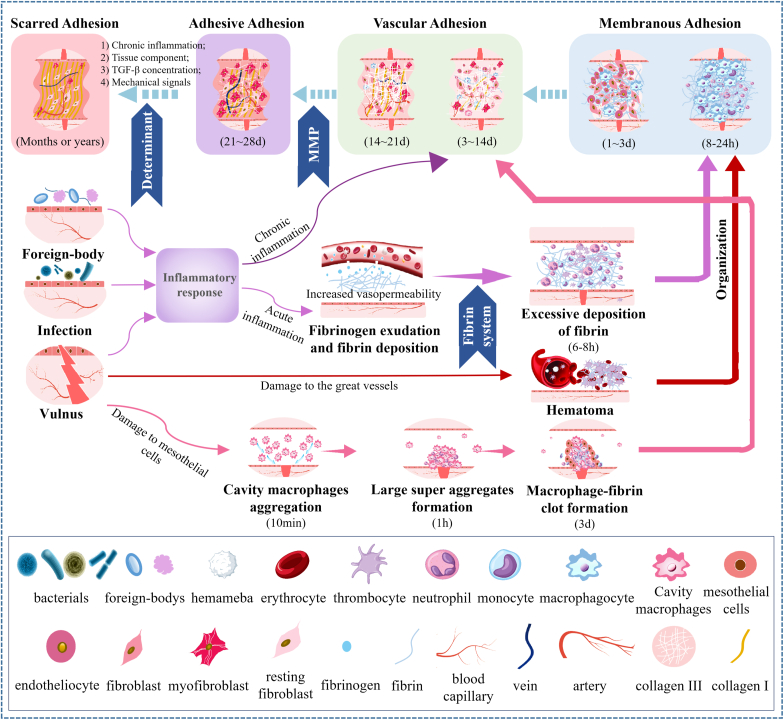
**The occurrence and development of POA.** Foreign-body, infection or vulnus trigger the subsequent inflammatory response, hematoma or cavity macrophages aggregation in different tissues. Among them, acute inflammations, especially fibrinous inflammation and serous inflammation, are easily occur in mucosa (*e.g.*, pharynx, larynx, trachea, intestine) and serosa (*e.g.*, pleura, peritoneum, and pericardium); Hematoma caused by damage to great vessels usually takes place in highly vascular tissues such as dura mater; Cavity macrophages easily aggregate in injured serosal membranes (*e.g.*, peritoneum and pericardium) in case of sterile injury. The excessive deposited fibrin, hematoma or macrophage-fibrin clot then play the role of cell scaffolds to attract cell invasion, excessive extracellular matrix (ECM) deposition and vascularization, as well as ECM remodeling, performing as “adhesion tissues” to interconnect the adjacent tissues or organs. According to the specific composition of adhesion tissues, we classified POA into four categories and named them as “membranous adhesion”, “vascular adhesion”, “adhesive adhesion” and “scarred adhesion”, respectively. The severity of injury and inflammatory response, as well as specific tissue characteristics together decide the occurrence, development and ending of POA in different tissues.

As it was reported, the pathophysiology of different kinds of adhesions is still not entirely understood. And, factors related to adhesion formation, such as local inflammatory reaction, infection, and oxidative stress are usually complicated [5,47,48]. In addition, pathophysiology for specific tissues exists differences. However, it could be found that all kinds of adhesions in different tissues had some similarities. For example, excessive deposition of fibrin, proliferation of fibroblasts/myofibroblasts and deposition of collagen and collagen fibers are regarded as important factors in adhesion formation. Basing on these similarities, we tried to abstract and outline the causes, progressions and outcomes of adhesions, and to find the determinants of the different developmental processes and outcomes in terms of causes and specific tissue sites (Fig. 1).

### 2.1 Adhesion classification according to the composition of adhesion tissues

POA is regarded as the consequence of abnormal tissue repair. This kind of response for organism to the outside disruption of internal balance usually starts when tissues injury, infection or foreign bodies occurs. Meanwhile, adhesion means that two adjacent tissues or organs are fused together by a certain medium, which could be tentatively defined as “adhesive tissue”. It could be found that adhesions usually take place over a period, during which the adhesive tissue changes gradually. For example, previous reports suggested that abdominal adhesions began to form on 3–5 days of post operation and then formed irreversibly dense adhesions within 7–14 days [49]; scarred adhesions occurring in epidural or tendon adhesion often need weeks or even months to develop [46,50].

Four kinds of adhesion tissues could be summarized based on the composition diversity of adhesion tissues and the previous classification of various postoperative adhesions [2,46,51,52]. They are (1) adhesive tissue composed of fibrin/hematoma clot and inflammatory cells (neutrophil), (2) loose connective tissue composed of mesothelial cells/fibroblasts/myofibroblasts, collagen and capillary vessels, (3) dense connective tissue composed of myofibroblasts/resting fibroblasts, collagen I, nerve fibers, arterial and vein vessels, (4) scarred tissue composed of collagen I and small number of myofibroblasts/resting fibroblasts. Hence, we tried to classify these adhesions into four types and named them as: “Membranous Adhesion”, “Vascular Adhesion”, “Adhesive Adhesion”, and “Scarred Adhesion”, respectively.

It can be found that the four types of adhesions have a degree of developmental relationship. For example, scarred adhesions develop from the adhesive and vascular adhesions. However, it is difficult to determine one tissue will necessarily develop into which type of adhesions, while we could identify the influencing factors that affect the occurrence and development of adhesions. The main components and influencing factors in each phase were summarized in Table 2.

**Table 2:** The main components of adhesion tissues during POA formation and the main influencing factors.

Different stages during POA formation	The main components	The main influencing factors	Consequences
0–8h	**Inflammatory cells:** [53] Neutrophils; Basophils; Mast cells; Eosinophils; Macrophages **Special for Serosa:** [54] Cavity macrophages **Special for tissues easy with abundant blood supply:** [55] Hematoma **Other components:** [56] Fibrinogen and fibrin	**The degree of injury and inflammatory:** [57,58] Chemokines: interleukin-6 (IL-6), interleukin-1β (IL-1β), and tumor necrosis factor-α (TNF-α); **The excessive formation and deposition of fibrin:** [56] Thrombin and thrombin activator; Thrombin inhibitor; Rough surface [59] **Insufficient fibrinolysis:** [[60], [61], [62], [63]] Proenzyme plasminogen; Plasmin; plasminogen activator (PA); Plasmin inhibitor (PI); Plasminogen activator inhibitor (PAI); The amount and activity of mesothelial cells **Special for tissues easy to form hematoma:** [55] Hematoma amount; Growth factors such as platelet derived growth factor, fibroblast Growth factor and transforming growth factor beta (TGF-β) **Special for serosa:** [54] Amount and aggregation of cavity macrophages: Macrophage scavenger receptor 1 (MSR1); Macrophage receptor with collagenous structure (MARCO); Purinergic receptor P2X7; CD44	Membranous adhesion
8–24h	**Inflammatory cells** [53]: Macrophages; Neutrophils **Special for serosa:** [64,65] Mesothelial cells; Cavity macrophages **Other components:** [58] Fibrin clot/Hematoma clot/Macrophage-fibrin clot		
1-3d	**Inflammatory cells** [53]: Macrophages; Neutrophils **Repairing cells:** [66] Fibroblasts; Endothelial cells **Special for serosa:** [64,65] Mesothelial cells **Other components:** Fibrin clot/Hematoma clot/Macrophage-fibrin clot		
3-14d	**Repairing cells:** [67] Fibroblasts; Endothelial cells **Inflammatory cells** [53]: Macrophages **Special for serosa:** [68] Mesothelial cells; Myofibroblasts derived from mesothelial to mesenchymal transition (MMT) **Other components:** [69] Collagen Ⅲ; Blood capillary	**The excessive formation of ECM:** [[70], [71], [72], [73]] Chemokines secreted by inflammatory cells and injured cells (*e.g.* platelet derived growth factor (PDGF), fibroblast growth factor (FGF), TGF-β, IL-1, IL-6, TNF-α, and VEGF) recruiting fibroblasts, mesothelial and endothelial cells, and triggering their proliferation and differentiation. **Dysregulation of ECM degradation:** [[74], [75], [76]] Matrix metalloproteinases (MMPs); Inhibitors of MMPs: tissue inhibitor of matrix metalloproteinases (TIMPs) and α2-macroglobulin	Vascular adhesion
14-21d	**Repairing cells:** [67,77] Fibroblasts; Endothelial cells; Myofibroblasts derived from fibroblasts **Inflammatory cells** [53,78]: Macrophages **Special for serosa:** [68] Myofibroblasts derived from MMT **Other components:** [69,79] Collagen Ⅲ; Arteriole; Venule; Capillary vessels; Collagen Ⅰ		
21-28d	**Repairing cells:** [77,80,81] Myofibroblasts; Resting fibroblasts; Fibroblasts; Endothelial cells; **Inflammatory cells** [53,78]: Macrophages **Other components:** [82,83] Type I collagen fibers; Blood vessels; Capillary vessels; Nerve	**Resources of myofibroblasts transition/tissue components** [77] **Factors Promoting myofibroblasts transition:** **Chemical signals:** [[84], [85], [86]] TGF-β (especially TGF-β1); ED (extradomain)-A fibronectin (EDA FN) **Mechanical signals:** [[87], [88], [89], [90]] Stiffening and/or straining of the ECM; The increased stiffness; Caveolin1 and yes-associated protein (YAP1)	Adhesive adhesion
Months or years	**Repairing cells:** [80,81] Resting fibroblasts; Myofibroblasts; **Inflammatory cells** [78]: Macrophages **Other components:** [82,83] Type I collagen fibers; Blood vessels; Nerve	**Chronic inflammation inducers:** [73,78,91,92] Foreign bodies; Hypoxia; Reactive oxygen species (ROS) **Resources of myofibroblasts transition/tissue components** [77] **Factors Promoting myofibroblasts transition:** **Chemical signals:** [[84], [85], [86]] TGF-β (especially TGF-β1); ED (extradomain)-A fibronectin (EDA FN) **Mechanical signals:** [[87], [88], [89], [90]] Stiffening and/or straining of the ECM; The increased stiffness; Caveolin1 and yes- associated protein (YAP1)	Scarred adhesion

### 2.2 Basis of adhesions/slight adhesions: excessive deposition of fibrin, hematoma or macrophage-fibrin clot

Excessive deposition of fibrin has been suggested as a common direct cause of adhesions in majority of soft tissues. For peritoneal adhesion, the fibrin band or bridge formed by fibrinous matrix coating on two peritoneal surfaces was demonstrated as the base of organized adhesions by simply apposing two peritoneal organs [93]. And, permanent adhesion characterized by collagen deposition and vascular ingrowth was suggested to form from the fibrin matrix invaded by fibroblasts and vessels [83]. Homoplastically, the cause of pericardial adhesions was also summarized to be attributed primarily to the stripping of pericardial mesothelial cells during surgery and the subsequent adhesion and accumulation of fibrin at the site without mesothelial cells [34]. In addition, the vast deposition of fibrin was also reported to cause adhesion between the tendon and peripheral tissue [34]. For intrauterine adhesions, similar conclusion has been reported that insufficient fibrinolysis led to the fibrous tissue via the invasion of fibroblasts and the appearance of angiogenesis [94,95].

Its effects on adhesions include three aspects: (1) The over-deposited fibrins could connect the adjacent tissues or organs because of their own high adhesiveness; (2) Binding to fibroblasts, endothelial cells, smooth muscle cells, leukocytes, etc. through cell surface integrin receptors, thus serving as a scaffold for cell growth and proliferation or a matrix for wound healing [96]; (3) Producing cytokines, such as IL-6, IL-8, monocyte chemotactic protein-1 (MCP-1) and VEGF, by inducing cells, such as peritoneal mesothelial cells and macrophage, to promote inflammatory responses and exacerbate adhesion formation [93,[97], [98], [99]].

The excessive deposition of fibrin is related to two aspects of causes: (1) The excessive formation of fibrin; (2) Insufficient fibrinolysis [60]. Hence, it was reported that the imbalance between the formation and degradation of fibrin induced adhesions [56].

**Fig. 2:**
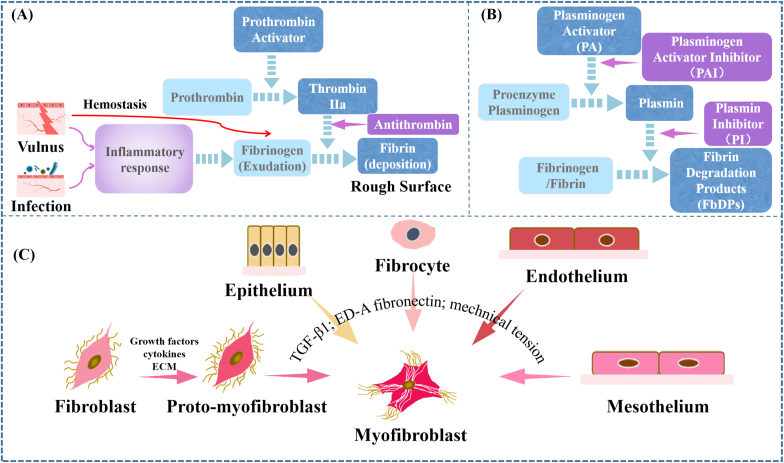
**Schematic diagrams of some mechanisms during POA formation.** (A) The formation of fibrin. (B) The degradation of fibrinogen/fibrin. (C) The resources of myofibroblast.

Fibrin is formed during hemostasis and acute inflammation [58]. Regardless of which way it is formed from, the formation of fibrin is the result of activation of the coagulation cascade (Fig. 2A). That is to say, fibrinogen permeating from or flowing out the blood vessels would be converted into insoluble fibrin monomers under the action of thrombin. And, these fibrin monomers then interact with one another and further polymerize to form a fibrin band [58].

Large amounts of fibrinogen in the inflammatory exudate are the consequence of heavily damaged capillaries and small veins, and the subsequent significant increase of vascular permeability [57]. In the early stages of milder injury, the exudation is dominated by globulin and albumin with small molecular weight. In addition, fibrinous inflammation and serous inflammation were reported to exude fibrinogen. Among them, fibrinous inflammation, characterized by high fibrinogen content in the exudate, is mostly caused by certain bacterial toxins or a variety of endogenous and exogenous toxins, and often occurs in the mucosa, serosa and lungs [100]. Excessive fibrinogen permeating in fibrinous inflammation was reported to be easily deposited and remodeled, ultimately evolving into fibrosis. Pericardial adhesion triggered by pericarditis and peritoneal adhesion triggered by bacterial peritonitis are good examples [101,102]. Serous inflammation often occurs in the serosa, mucosa and loose connective tissue with small amount of fibrinogen in exudate. However, severe serous inflammation could develop into fibrinous inflammation and even cause adhesions [103]. Hence, it can be hypothesized that the high probability of adhesion formation in plasma membranes, such as pericardium, pleura, peritoneum, ectocardium, myocardial ectocardium, and intestinal ectocardium, as well as mucous membranes, such as endometrium and myocardial endocardium might be associated with easy occurrence of the two types of inflammation.

Consequently, increasing the degree of injury and inflammatory could promote the exudation of fibrinogen. And, increasing thrombin and thrombin activator formation or decreasing the amount of thrombin inhibitor could contribute to excessive deposition of fibrin, thereby triggering adhesions. Besides, there is no doubt that a rough surface is an essential condition for fibrin deposition. It was reported that the denudation of the pericardial mesothelial cell (PMC) lining was a key factor for the subsequent intrapericardial adhesion formation because it provided denuded area for the adhesion of fibrin, platelets, and inflammatory cells [59].

Appropriate fibrin deposition as one part of the physiological protective mechanism against invading microorganisms plays a temporary role in tissue repair. And, it is usually degraded thoroughly during the structure and function restoration in normal tissue. The degradation of fibrin is regulated by the fibrinolytic system. Under the effect of plasmin, the fibrinogen or fibrin could be degraded into fibrin degradation products (FbDPs) [104]. Active plasmin needs to be converted from the inactive proenzyme plasminogen by plasminogen activator (PA) including tissue plasminogen activator (tPA) and urokinase plasminogen activator (uPA) [105]. Among them, tPA is reported as the principal activator of plasminogen [61]. Hence, the amount of proenzyme plasminogen and the activity of plasmin and PA are important for the degradation of fibrin/fibrinogen (Fig. 2B).

However, the plasmin activity could be inhibited by plasmin inhibitor (PI) such as α2-antiplasmin, which also has high affinity for plasminogen and fibrin to prevent premature lysis of the fibrin clot [62,63]. And, the plasminogen activator inhibitor (PAI) 1 could also inactivate uPA and tPA by forming inactive uPA-PAI-1 and tPA-PAI-1 complexes [[106], [107], [108]]. Hence, the imbalance between PA and PAI might cause excessive deposition of fibrin and subsequent adhesion. It was reported that the levels of PAI-1 and tPA-PAI-1 complex in peritoneal tissue samples were prominently increased for patients with severe adhesions compared with those with light adhesions [109]. In addition, fibrinolytic insufficiency was also suggested as a cause of adhesion formation in a study [110].

Consequently, the whole factors that could decrease the amount or inhibit the activity of PA and plasmin and increase the amount or the activity of PAI and PI may prevent the sufficient degradation of fibrin and fibrinogen, promoting the formation of adhesions. PA is released by mesothelial and endothelial cells [105]. And, the PAI-1 is produced and released by various cells including endothelial cells, mesothelial cells, platelets, macrophages and fibroblasts [111]. As the main cells releasing PA and PAI, it is no doubt that the damaged and regenerated mesothelial cells might be a key factor of adhesion formation. In addition, surgery and inflammation might play an important role. It was reported that the overexpression of p65 protein led to excessive release of inflammatory cytokines TNF-α, which is quite important for adhesion formation because it can not only promote blood coagulation and inflammation but also reduce fibrinolysis by stimulating the release of PAI and inhibiting the production of PA in peritoneal cavity [112].

Except for excessive fibrin, hematoma was also reported to easily recruit cells as scaffold for cell growth and proliferation. Songer et al. [113] suggested that hematoma might be the initial stage of scar formation. Different from fibrin in the inflammatory exudate, hematoma usually occurs when large blood vessels rupture under external force. Except for fibrin, blood compositions such as blood platelet, hemameba, albumin, growth factors are composed in hematoma clot. And, growth factors such as platelet derived growth factor, fibroblast growth factor and transforming growth factor beta (TGF-β) released from hematoma clot could promote the proliferation and differentiation of fibroblasts, promoting adhesion formation [55]. As it was reported, adhesions caused by hematoma appear in epidural adhesions might be related to the abundant blood supply to dura and surrounding tissues. However, it is still controversial about the relationship between hematoma and epidural adhesions. Toulialos et al. [114] pointed out that soft tissue trauma with a combined hematoma could cause peridural fibrosis and adhesions regardless of the location of the spinal canal. Whereas Christian et al. [115] found that complete removal of the hematoma also couldn't completely prevent scar adhesion formation, but only reduce the degree of scar denseness. Meanwhile, some studies found that the increased vascular permeability and inflammatory response caused by surgical trauma or other causes resulted in epidural adhesions [116,117]. Even though a causal relationship between hematoma and adhesion is difficult to be established, it is obvious that the increase of hematoma amount would increase the severity of adhesions. Since fibrin is the main reason of blood agglutination, the degradation of hematoma also depends on fibrinolytic system.

Besides, it's worth noting that resident cavity GATA6^+^ macrophages have been reported to play important role in serosal adhesion forming in case of sterile injury [54]. Resident cavity GATA6^+^ macrophages, characterized by the expression of CD102 (Icam2), high levels of F4/80 and the transcription factor GATA6, are one kind of coelomocytes in coelomic cavities [118,119]. They can clear bacteria via phagocytosis or through inducing intra-abdominal formation of fibrin clots to immobilize bacteria [120]. More importantly, these GATA6^+^ macrophages can rapidly form aggregates at the injury site of the peritoneal membrane in response to damage associated molecular patterns (DAMPs), the immunostimulatory molecular patterns inducing inflammation in sterile injury. This interaction relies on different receptor molecules such as macrophage scavenger receptor 1 (MSR1), macrophage receptor with collagenous structure (MARCO), purinergic receptor P2X7, and CD44. Dysregulation of the aggregation of these macrophages on mesothelial surfaces will occur in case of large injuries, which has been regarded as precursors to adhesions [54]. Zindel et al. [54] found that the amount and intensity of peritoneal adhesions in a mouse model could be significantly reduced by depleting peritoneal cavity macrophages or inhibiting their aggregation. Even though the aggregation of macrophages was demonstrated to be largely independent of fibrin crosslinking, serosal adhesion after sterile injury is usually related to both macrophage aggregation and fibrin deposition. It is the macrophage-fibrin deposit that acts as the scaffold for the subsequent inflammatory cells, endothelial cells, mesothelial cells and fibroblasts/myofibroblast.

Excessively deposited fibrin/blood/macrophage-fibrin clot along with a large amount of neutrophil are the reactions of organism to operative trauma and acute inflammation during 6–8 h after surgery [53]. Subsequently, monocyte or macrophagocyte would invade the clot. For serosa, mesothelial cells would be also recruited. In addition, under the effect of growth and chemokines such as interleukin-6 (IL-6), interleukin-1β (IL-1β), and tumor necrosis factor-α (TNF-α), fibroblast and endotheliocyte will be recruited and secret extracellular matrix to gradually replace the clot [70,71].

In most cases, the mixture of fibrin/hematoma/macrophage-fibrin clot, inflammatory cells, and small amount of other compositions were only regarded as the basis of adhesions. In some cases, the mixture with a certain adhesive force to connect the adjacent tissues or organs could result in a slight adhesion, which is mild enough to be separated by tearing and pulling (i.e., blunt separation) [121]. Hence, we named this adhesion tissues during organization progress that the depositional clot is gradually replaced by granulation tissues as “Membranous Adhesion” [122].

### 2.3 Development of adhesions: excessive deposition of extracellular matrix with vascular invasion

As it was reported, adhesion formation is a dynamic but dysregulated regenerating tissue repair process [66]. Fibroblasts, as the main cells of tissue repair, usually appear in the defect area in 2–3 days after surgery under the chemotaxis of inflammatory mediators and growth factors, and then grow into the depositional clot together with capillaries. Particularly, mesothelial cells in the surface of serosa would also cover the macrophage-fibrin clot and further translate into myofibroblasts [64,65]. Stimulated by growth factors, such as platelet derived growth factor (PDGF), fibroblast growth factor (FGF), TGF-β, IL-1 and TNF, fibroblasts and myofibroblasts proliferate and begin to produce extracellular matrix (ECM) [67]. In addition, collagen formation (collagen type Ⅲ is the main composition) would be most active during the following 1 week and be minimal after 3 weeks [69]. Meanwhile, the capillary gradually occurs and further develops into small arteries and veins. And, the nerves gradually grow into tissues. Because macrophages, neutrophils and capillary endothelial cells in granulation tissue can phagocytose bacteria and tissue debris, as well as break down necrotic tissue and fibrin by releasing various protein hydrolases, the deposited fibrin/blood clot would gradually disappear [69].

The outcome of wound healing or impairment (*e.g.,* adhesion formation) largely depends on the extent of cellular migration, proliferation and ECM turnover. And, it was suggested that adhesion formation was usually associated with the excessive deposition of ECM, which mainly depended on the balance between formation and degradation of ECM [74,75].

Fibroblast/myofibroblasts are the main kinds of cells responsible for the synthesis of ECM. It was reported that fibroblasts played a key role in epidural adhesion after laminectomy [123]. Meanwhile, Xiang et al. [124] found that most of fibroblasts in peritoneal adhesions were myofibroblasts identified as α-smooth muscle actin (SMA)-positive. Foster et al. [1] also found that fibroblasts within adhesions in patients and mouse models expressed fibroblast markers, such as α-SMA, vimentin, and collagen 1 (COL1), and platelet derived growth factor receptor alpha (PDGFRA), a known promotor of systemic fibrosis [125].

Fibroblasts in wound healing are mainly derived from local fibroblasts, undifferentiated mesenchymal cells, and perivascular fibroblasts [77]. And, when visceral injury occurs, fibroblasts involved in the repair process are mostly from the interstitium, capsule, and submucosal or subserosal connective tissue. And, myofibroblasts in peritoneal adhesions has been demonstrated to mainly arise from mesothelial to mesenchymal transition (MMT). Specifically, Fischer et al. [64] used an inducible genetic lineage tracing system based on the mesothelial cell marker Procr to indicate that adhesion myofibroblasts were not derived from fibroblasts, but from mesothelial precursors. And, Joel et al. [126] used a Wt1-based genetic lineage tracing to show the same finding. Furthermore, they demonstrated that the activation and *trans*-differentiation of mesothelial cell niche were driven by EGFR-signaling. And, fibrin was also regarded as an inducer of MMT [68].

As fibroblasts/myofibroblasts migrate under the chemotaxis of chemokines secreted by inflammatory cells and other injured cells, severe inflammatory reaction and trauma would promote the number of cells in the injured site. Meanwhile, the proliferation of fibroblasts/myofibroblasts could be promoted by growth factors released from platelets and macrophages. Overproduction of proinflammatory cytokines, such as TNF-α and IL-6, could also trigger the proliferation of fibroblasts/myofibroblasts and collagen synthesis [72,73]. Thus, the increase of injury and inflammatory severity would active cell proliferation and ECM deposition.

The degradation of ECM is mainly controlled by matrix metalloproteinases (MMPs) including collagenase (MMP 1 and 13), gelatinases (MMP 2 and 9) and stromelysin (MMP 3). Among them, MMP-1 can degrade collagen types Ⅰ，Ⅱ，Ⅲ，Ⅶ and Ⅹ, and can be activated by MMP-3 or stromelysin-1 under *in vitro* conditions [76]. MMP-2 and MMP-9 can degrade collagen type Ⅳ. MMP-3 can degrade collagen types III, IV and IX, elastin, fibronectin, versican, aggrecan, and laminin, *et* [127]. The expression of MMP in normal tissue is usually low, while it would be increased in various pathological conditions, such as various of connective tissue disorders, inflammation, impaired and nonhealing wounds, as well as extensive remodeling [128]. Furthermore, the proteolytic activity of MMPs is controlled by tissue inhibitor of matrix metalloproteinases (TIMPs, including TIMP-1 to TIMP-4) and through zymogram inhibition by α2-macroglobulin [129]. Besides their crucial role as the inhibitors of MMPs, TIMPs are also reported to affect cell growth and angiogenesis [130]. Hence, the unbalance between the expression and activity of MMPs and TIMPs could result in impaired wound healing and even tissue adhesions [131,132]. Previous studies showed that concentration of MMP-9 in peritoneal fluid is significantly lower in women with pelvic adhesions compared to those with a normal pelvis [133,134]. A similar result was also presented in the study of Nasser et al. that an inverse relation between TIMP-1 and MMP-1 levels in serum and peritoneal fluids was found in subjects with moderate and extensive adhesions [135]. Except for that, the expression levels of TIMP-1 mRNA and protein among the serosal tissue of intraperitoneal organs and adhesions exceeded those of MMP-1 by 100- to 10,000-fold and by 2- to 10-fold, respectively [136].

Hence, the proliferation of fibroblast/myofibroblast, the decrease of the expression and activity of MMPs, as well as the increase of the expression and activity of TIMPs would elevate the probability of adhesions.

New vascularized tissues formed during this proliferation period gradually fill the wound defect and replace the depositional clot. It is mainly composed of unorganized collagen type Ⅲ with irregular structure, performing low crack strength [69]. However, the vascularized tissues are stronger than membranous adhesion tissues, and often need to be separated by knife or scissors (i.e., sharp separation). Therefore, we named this kind of vascularized tissues as “vascular adhesion”.

### 2.4 Mature of adhesions: abnormal remodeling of the extracellular matrix

During abnormal tissue repair, the anabolism of collagen tends to be stable at about four weeks after surgery, and collagen fibers would gradually change from original disorderly arrangement into neat and orderly bundles, with increased proportion of collagen type Ⅰ [79]. Moreover, the number of blood vessels and cells gradually decreases with active fibroblasts increasingly replaced by myofibroblasts or resting fibroblasts. And, the adhesion tissues may become pain-sensitive because of the ingrowth of nerve fiber [82]. This kind of adhesion tissues firmly attaches to adjacent tissues or organs with outstanding mechanical properties. Hence, we named it “adhesive adhesion”.

When there exists chronic inflammation, adhesive tissues could develop into scarred tissues in months or years after surgery [137]. The scarred tissues consist almost exclusively of thick type I collagen fibers, a little of resting fibroblasts, myofibroblasts and blood vessels, and usually contract and pull the surrounding tissues, causing great damage to the body. Hence, we named this kind of adhesion “scarred adhesion” [83].

The abnormal remodeling of ECM could also be regarded as fibrosis process, during which myofibroblasts play a vital role because the collagen synthesized by them is four to five times than that by fibroblasts and the collagen I is mainly synthesized via α-SM actin-expressing myofibroblasts [80,81]. During normal wound repair, myofibroblasts are transiently present to promote wound contraction and connective tissue repair. However, myofibroblasts persist in fibrotic lesions, causing excessive accumulation of ECM and remodeling of tissue structure and resulting in the change of ECM from normal type III and IV collagen-based to type I collagen-based [138].

The source of myofibroblasts during tissue repair apart from serosa repair is respectively complex. It was reported that 50% of myofibroblasts arise from local resident fibroblasts through proliferation, 35% of myofibroblasts derive via differentiation from bone marrow, 10% of myofibroblasts arise through the endothelial-to-mesenchymal transition program, and 5% of myofibroblasts derive through the epithelial-to-mesenchymal transition program [77]. (Fig. 2C) Therefore, it can be speculated that the surgical site with more myofibroblasts sources would be easier to form scarred adhesion. This might be one reason why dura mater, which is mainly composed of fibroblast and collagen fibers, are more prone to form scarred adhesion [139].

Both fibroblast-to-myofibroblast differentiation and MMT are regulated by mechanical and chemical signals, of which TGF-β is a key factor [140,141]. TGF-β consisting of TGF-β1, 2, and 3 is the main inducer of the myofibroblastic phenotype, and is able to upregulate collagen and α-SM actin expression in fibroblasts both *in vitro* and *in vivo* [84,85]. Much data indicated that the dysregulation of TGF-β production might cause tissue fibrosis [142]. Previous studies showed that adding TGF-β to the adhesion model increased the severity of the adhesions. And, TGF-β could also promote the formation of abdominal adhesions after surgical injury to the uterine horns [143]. Compared with TGF-β2 and 3, TGF-β1 plays a more important role in scar tissue formation [144]. Lucas et al. [144] found that antibodies to TGF-β1 inhibited scarring, while a panspecific antibody to TGF-β1, 2, 3 and antibodies to TGF-β2 did not affect scar formation. Whereas TGF-β2 can upregulate the production of TGF-β1 by fibroblasts, monocytes, and macrophages [145]. For TGF-β1, it leads to fibrosis mainly through the TGF-β1/Smad signal pathway that Smad3 and Smad7 jointly regulate TGF-β1 signal transduction [146]. Specifically, TGF-β1 can directly induce the production of ECM via activating Smad3, thereby improving fibrosis. Nevertheless, Smad7 can block the phosphorylation of Smad3 through ubiquitin ligase to degrade TGF-β1 receptor (TβRI) or by binding TβRI, thus inhibiting the activation of Smad3 and blocking TGF-β1 signal transduction [147,148]. In addition, ED (extradomain)-A fibronectin (EDA FN) is essential for the effect of TGF-β1 on fibroblast differentiation. Because the expression of TGF-β1 gene and the increase of collagen type I and actin mRNA induced by TGF-β1 have been demonstrated to depend on the EDA FN-driven signaling [86].

Besides the effect of TGF-β, mechanical signals are also necessary. On the one hand, TGF-β1, which is present in the ECM as a large latent complex including latent TGF-β1-binding protein and latency-associated peptide, needs to be liberated by proteolytic enzymes or integrin-dependent mechanically induced mechanisms [87]. That is to say, stress fibers exert force to free TGF-β1 through transmembrane integrin. In addition, stiffening and/or straining of the ECM can increase the availability of TGF-β1 [149,150]. On the other hand, the early transformation of fibroblasts into proto-myofibroblasts was reported to depend on the mechanical changes occurring to the wound, especially the increased stiffness [88]. And, the strained ECM can maintain a feedback mechanism to ensure a persistent fibrotic activity of myofibroblast [151]. Moreover, Philip et al. [90] further proved that tensile forces drove the reversible fibroblast-to-myofibroblast transition during tissue growth in engineered clefts. For MMT, exposing mesothelial cells to cyclic mechanical forces increased MMT in experimental human and murine models. The biomechanical induction of MMT was reported to be driven by Caveolin1 and yes-associated protein (YAP1) [89]. Hence, stretching the wound or splinting can result in increased mechanical loading, which in turn increases myofibroblast activity and leads to increased scar formation. This might be one reason why scarred adhesions take place at the intrauterine and peritoneum where have harsh mechanical environments with a high probability [83].

Consequently, the increase in the amount and activity of TGF-β, fibroblast-to-myofibroblast differentiation and myofibroblast proliferation, as well as tensile forces on the ECM could promote the probability of scarred adhesion formation. And, the synthetic actions of these factors in the injured site decide which kind of adhesion would ultimately format.

### 2.5 Chronic inflammation: the key factor during adhesion formation

In fact, adhesions including “vascular adhesion”, “adhesive adhesion” and “scarred adhesion” develop from hemostasis and acute inflammatory exudate, which are triggered by surgical trauma, infection, etc. Usually, they would undergo a transition to chronic inflammation. As reported, fibrosis was considered the irreversible end stage of chronic inflammation, and recurrent inflammation is an inevitable process in the development of fibrosis [91]. Moreover, chronic nonspecific inflammation was reported to lead to arachnoiditis, a potential cause of post-laminectomy neurological deficits [152]. The occurrence and development of chronic inflammation are related to many factors, of which foreign bodies, hypoxia and the subsequent oxidative stress require special attentions.

Firstly, foreign bodies left over from the surgery, such as cotton fibers and autologous bone fragments, can promote scar proliferation by triggering a foreign body reaction to encapsulate the foreign body, which may cause adhesions. Hoyland al et [78]. studied the impact of retained surgical swab debris in post-laminectomy arachnoiditis and peridural fibrosis and found that the introduction of foreign material might have a role in the pathogenesis of postoperative periradicular fibrosis.

Besides, it is worth noting that hypoxia may play an important role in adhesion formation because of its effect on fibrin, ECM and growth factors related to fibroblasts and mesothelial cells [92]. Concretely, hypoxia was reported to induce proliferation while inhibit apoptosis in fibroblasts [153]. In addition, it could increase the expression of PAI-1 and TIMP-1 in normal and adhesion fibroblasts and peritoneal tissues, and increase VEGF production via activation of hypoxia-inducible factor (HIF)-1α in normal and adhesion fibroblasts, animal adhesion tissues, and human adhesion mesothelial cells [154,155].

In addition, tissue hypoxia may lead to increased oxidative stress and increase the production of nitrogen and oxygen free radicals, resulting in DNA damage and increased production of oxidized proteins and exacerbating chronic inflammation [73]. During the first 5 min after hypoxia, free radicals were demonstrated to produce obviously by increasing the formation of reactive oxygen species (ROS) [156]. These free radicals can promote the expression of many factors, including TGF-β, IL-6, collagen type I and VEGF [157]. Meanwhile, oxidative stress damage is also associated with the activation of MMP. Specifically, ROS can induce the activation of transcription factors, activating the activity of MMPs. Clinical findings showed that the relative level of MMP was positively correlated with the degree of oxidative stress [158]. Besides, hypoxia can induce the production of superoxide, fibroblasts exposed to which would produce pro-fibrotic factors, such as TGF-β and collagen type I [159]. Meanwhile, the expression of collagen-1 mRNA and TGF-β could also be increased in hypoxia conditions in cultured human peritoneal fibroblasts and mesothelial cells [160].

Hence, the severity increase of foreign body reaction, hypoxia and oxidative stress would exacerbate chronic inflammation, promoting the probability of adhesion formation.

## 3 Possible strategies for preventing adhesion by using biomaterials and the existing practices

According to the causes and development of adhesions in the second part, it can be found that minimizing the damage to the cells or blood vessels in operation area through “minimally invasive surgery”, adequately removing the residual foreign bodies such as debris, blood clots and surgical debris, sufficiently stopping bleeding, and reducing inflammation can minimize the occurrence of adhesions from the source [20]. However, because of the long period of adhesion formation, whether in acute inflammation or late chronic inflammatory process, it is necessary to intervene with effective biomaterials throughout the process.

For accessible surgical operation and proper anti-adhesive efficacy, ideal anti-adhesion biomaterials need to meet some basic requirements such as satisfactory biocompatibility and biodegradability to avoid triggering or exacerbating inflammation and immune response, enough retention time to pass the whole stage of adhesion formation, appropriate mechanical properties and adhesive force to prevent the deviation from a specific position and the breach of barrier integrity, *etc*. In general, anti-adhesion materials need to be degradable with a degradation time of 2–4 weeks to get over the inflammatory and abnormal proliferation period.

**Fig. 3:**
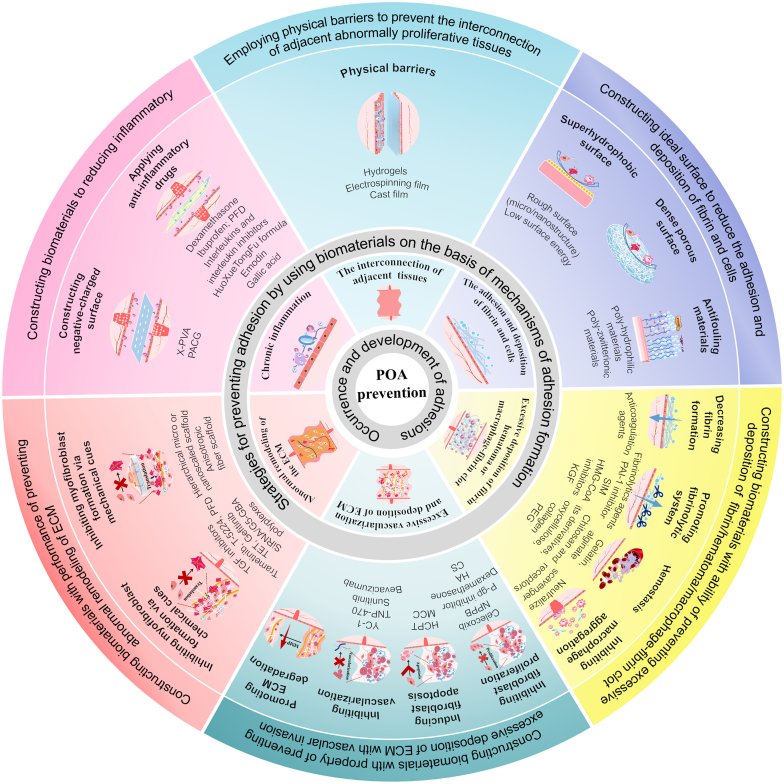
**Strategies for preventing POA by using biomaterials based on mechanisms of adhesion formation**. Six strategies could be proposed according to the corresponding determinants during the occurrence and development of POA. In addition, the combination of several strategies to construct multi-functional materials could be effective.

In addition, to prevent postoperative adhesion of soft tissues, biomaterials must prevent the occurrence and development of adhesion. Therefore, the following strategies could be proposed based on POA formation mechanisms: (1) employing physical barriers to prevent the interconnection of adjacent abnormally proliferative tissues; (2) constructing an ideal surface to reduce the adhesion and deposition of fibrin and cells; (3) constructing biomaterials with the ability of preventing excessive deposition of fibrin/hematoma/macrophage-fibrin clot; (4) constructing biomaterials with the property of preventing excessive deposition of ECM with vascular invasion; (5) constructing biomaterials with the performance of preventing abnormal remodeling of ECM; (6) constructing materials to reduce inflammatory; (7) combining several strategies (Fig. 3).

### 3.1 Employing physical barriers to prevent the interconnection of adjacent abnormally proliferative tissues

Since tissue adhesions rely on the interconnection between adhesion tissues and the adjacent tissues, it will be effective to prevent the adhesion formation by employing physical barriers, which can simply and effectively separate the neighboring injury tissues to avert the interconnection of adjacent abnormally proliferative tissues [45]. Hence, physical barrier is still the most widely used method for post-adhesion prevention in clinical. Ideal physical barriers need to easily adhere to the wound, be biocompatible and biodegradable, possess the appropriate mechanical strength, and prevent the penetration of cells without impeding nutrients diffusion (dense porous with pore size smaller than cell diameter (the eukaryotic cell diameter ranges from 10 to 100 μm) [161]) [162,163].

Hydrogels, electrospinning film, sponge [[164], [165], [166]], microparticle [167,168], casting film [169], *etc*. have been widely applied as physical barriers. Among them, electrospinning film and hydrogels are applied wider compared to sponge, microparticles and casting film because they are easier to form bidimensional membrane with appropriate mechanical property. And, microparticles are prone to be used with electrospinning film and hydrogels. The advantages, disadvantages, and suitable application directions of different material forms are summarized in Table 3.

**Table 3:** The advantages, disadvantages, and suitable application directions of different types of material for POA prevention.

Material types	Advantages	Disadvantages	Suitable application directions	References
Electrospinning film	1 Easily regulated fiber diameter and pore size to prevent cell crossing; 2 Controllable fiber orientation to construct micropatterned surfaces; 3 Easily functionalized to achieve multifunction; 4 Good degradation property for a long-time POA prevention; 5 Good mechanical property for harsh biomechanical environment and tissues with a large area of damage; 6 Low swelling; 7 Appropriate for a wide range of polymers;	1 Probable organic solvent remains; 2 Difficult to prepare; 3 Difficult to meet the requirements of irregularly injured tissues to cover the entire surface; 4 Not suit for minimally invasive surgery	1 Cerebral dura mater; 2 Spinal dura mater; 3 Peritoneum; 4 Endometrium; 5 Pericardium	[[170], [171], [172]]
Hydrogels	1 Suit for irregularly injured tissues; 2 Suit for minimally invasive surgery; 3 Good biocompatibility due to its water content; 4 Easy to adhere to tissues; 5 Easy to prepare; 6 Easy to load drugs and achieve controlled-release	1 Easy to form tissue tamponade or mechanical compression because of significant swelling ratio; 2 Relatively poor mechanical and degradation properties; 3 Possible cytotoxicity when chemically cross-linked;	1 Tendon; 2 Peritoneum; 3 Spinal dura mater; 4 Pericardium; 5 Endometrium	[6,[173], [174], [175]]
Sponge	1 Easy to prepare; 2 Relatively good mechanical property; 3 Quickly stop bleeding; 4 Easy to adhere to tissues;	1 Difficult to control the pore size; 2 Easy to form tissue tamponade or mechanical compression because of significant swelling ratio;	1 Peritoneum; 2 Spinal dura mater; 3 Pericardium; 4 Endometrium	[[164], [165], [166]]
Microparticle	1 Stable drug/active biomolecules loading and allow their controlled release in a long time; 2 Easy to apply in clinical (subcutaneous or intramuscular injection);	1 Difficult to immobilize in specific injury site; 2 Difficult to play the role in cell barrier;	1 Peritoneum; 2 Tendon; 3 Endometrium	[167,168]
Casting film	1 Easy to prepare; 2 Smooth and dense and suit for cell barrier; 3 Easy to control the thickness	1Usually terrible mechanic property and stability; 2Low porosity to inhibit nutrient exchange in cells	1 Cerebral dura mater; 2 Spinal dura mater; 3 Endometrium	[169]

Electrospinning film exhibits superiority because of its special microporous structure and aspect ratio, good mechanical and degradation properties, easily functionalized performance, controllable porosity and pore size [170,171]. Nowadays, biodegradable synthetic polymers, such as poly(caprolactone) (PCL), poly(lactic-co-glycolic acid) (PLGA), polylactide (PLA), and etc. have been prepared into electrospinning film as a physical barrier [176,177]. Film products, including Seprafilm and Interceed, have been approved by FDA in the United States. However, effective hemostasis and suture to fix the film on the injured tissue surface are required during their application process. In addition, they are difficult to meet the requirements of irregularly injured tissues to cover the entire surface (*e.g.*, great vessels of the small intestine and heart). Hence, any uncovered intervening surfaces remain at risk of adhesion formation despite applying sheet-like film barriers [172].

Hydrogels mostly used for adhesion prevention are injectable hydrogels, which could be suit for all kinds of wounds because of their flexibility. However, a significant swelling ratio could result in tissue tamponade or mechanical compression, limiting their applications in some specific tissues such as cardiac, dura mater, *etc*. There are mainly three kinds of injectable hydrogels: (1) Physical hydrogels such as thermo-responsive hydrogel; (2) Chemical hydrogel crosslinked through Schiff base amino-aldehyde reactions [173], Michael addition thiol-ene reactions [178], or in situ polymerization; (3) Photo-crosslinked hydrogels. Even though there is no *in-vivo* chemical reaction or extra additives for physical hydrogel, several minutes are needed for the complete physical crosslink, which might increase the risk of infection. In addition, it has been reported that physical hydrogels degraded quickly (usually less than two days) because of their insufficient cross-linking, which vastly reduced their anti-adhesion efficacies [174]. For chemical hydrogel, chemical reactions usually apply at least two kinds of pre-gel solutions with enough time to completely cure, and the complicated operational process also presents a burden in surgical duration. Besides, the addition of chemical reagents might cause biocompatibility problems to a certain extent. For photo-crosslinked hydrogels, safety risks from the *in-vivo* ultraviolet irradiation must be taken into consideration.

Various biomaterials, such as chitosan and derivative [179], hyaluronic acid(HA) [180,181], poly(ethylene glycol) (PEG) [182,183], gelatin [184], dextran (DEX) and derivative [185], PEO [186], and etc., have been prepared into hydrogels and demonstrated their efficiency in reducing POA. However, some extent of adhesion could still be founded because of their low mechanical strength. Hence, some studies have been devoted to promoting mechanical properties to improve the adhesion prevention ability of hydrogels. For example, Yang et al. [6] developed tough supramolecular hydrogels using methylenediphenyl 4, 4-diisocyanate (MDI), imidazolidinyl urea (IU), and PEG through polyaddition. The multiple hydrogen bonds among IU and urethane groups enable the IU hydrogels to possess promising mechanical properties and adequate postoperative antiadhesion ability.

Even though providing physical barriers to prevent the interconnection of adjacent proliferative tissues could prevent adhesion formation to a certain extent, the adjacent proliferative tissues could exist as unconnected adhesion tissues and have a high probability of connecting when the barrier degrades partly or thoroughly. Hence, it is more effective to prevent the formation of abnormally proliferative tissues. Based on physical barriers, biomaterials also playing a role in preventing the formation of abnormally proliferative tissues need to be widely studied.

### 3.2 Constructing ideal surface to reduce the adhesion and deposition of fibrin and cells

To prevent the formation of abnormally proliferative tissues, the excessive deposition of fibrin, as the basis of adhesion formation, must be firstly prevented. Biomaterials may firstly avert the adsorption and deposition of fibrin and cells on the surfaces via adjusting surface structure or properties to form an anti-adhesive surface [187]. Preparing superhydrophobic surface and applying antifouling materials including poly-hydrophilic materials, poly-zwitterionic and self-deactivating biomaterials, have been widely used in POA prevention and indicated with satisfying effectiveness. In fact, the anti-adhesive surface can not only inhibit fibrin adhesion and deposition but also impede the adhesion of cells such as fibroblasts and endothelial cells during repair process, preventing the development of adhesion tissues.

#### 3.2.1 Constructing superhydrophobic surface

**Fig. 4:**
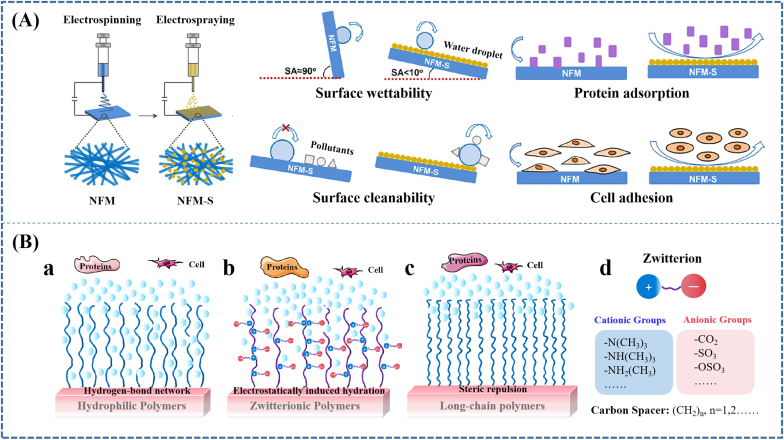
**Constructing ideal surfaces to reduce the adhesion and deposition of fibrin and cells, including constructing (A) superhydrophobic surface, (B) applying antifouling materials**. (A) Superhydrophobic surface fabricated by combining the hydrophobic fumed silica and electrospray deposition showed inhibiting effect on protein adsorption and cell adhesion; (B) Illustration of mechanisms how (a) poly-hydrophilic, (b) poly-zwitterionic polymers and (c) long-chain polymers prevent the protein and cell adhesion. Reproduced with permission: (A) [163] copyright 2020, Elsevier; (C) [188], copyright 2014, ACS.

Superhydrophobic surfaces with rough surfaces (micro/nanostructure) and low surface energy have drawn much attention because of prominent ability to prevent protein adsorption and cell adhesion [189,190]. Many methods, such as sol–gel [191], electrospray approach [163], layer-by-layer assembly [192], and chemicet alching [193] have been used to fabricate the superhydrophobic surface. For example, Mao et al. [163] fabricated a superhydrophobic surface for the physical barriers by combining the hydrophobic fumed silica (SiO_2_) and electrospray deposition, and found that the physical barriers with superhydrophobic surface showed good biocompatibility and obvious low-adhesion on protein and cells (Fig. 4A). Whereas their efficacy in preventing POA in animal model has not been demonstrated.

Even though these approaches performed superiority in constructing superhydrophobic surface, certain substrates, sophisticated and/or expensive processing machines, and strictly controlled reaction conditions limited their wide application. Hence, some efforts were devoted to using superhydrophobic materials. Qian et al. [194] prepared an electrospun DOX@bovine serum albumin/poly(ε-caprolactone)/MnO_2_ (DOX@BSA/PCL/MnO_2_) membrane. The membrane exhibited hydrophobic characteristics because of the hydrophobic PCL spinning, which had been demonstrated to play a crucial role in the significant spinal anti-adhesion property of membrane. Currently, we fabricated a triple-layered biocomposite for dura repair, the polylactic acid (PLLA) electrospinning film of which exhibited adhesion prevention ability because of its hydrophobic characteristics and physical barrier function [177]. Moreover, Zhou et al. [195] loaded Beeswax into poly-l-lactic acid by blending electrospinning. The incorporation of Wax into PLA increased the water contact angle of the PLA from 137.7 ± 3.40° to 154.8 ± 4.11°. Consequently, the beeswax (Wax)/poly-l-lactic acid (PLA) membrane with improved hydrophobicity achieved advanced peritendinous anti-adhesion outcome.

#### 3.2.2 Applying antifouling materials

Compared with constructing biomaterials with superhydrophobic surface, applying antifouling materials is the method used usually to prevent POA. Antifouling materials are well known for their outstanding ability to resist nonspecific protein adsorption and cell adhesion. There are three kinds of antifouling materials, including polyhydrophilic, polyzwitterionic, and self-deactivating materials. The polyhydrophilic materials such as PEG-based materials, polysaccharides and polyamides are characterized by electrically neutrality, hydrophilic nature, and hydrogen-bond acceptors/donor [196,197]. And the polyzwitterionic materials contain both anionic and cationic groups, and have high dipole moments and highly charged groups, whereas are still charge neutral [198,199]. The self-deactivating materials achieved antiadhesive property through chemically blocking the activity of the cell attachment-promoting components [200].

Since protein adsorption would be accelerated by reducing the free energy barrier arising from dehydration entropic effects, during which the expulsion of water molecules from both protein and material surface is the first and obligatory step [201]. Hence, strong hydration has been indicated as the main source of the non-fouling ability for both polyhydrophilic and polyzwitterionic materials because the hydration layer near surface plays as the physical and energetic barrier to prevent protein adsorption [202,203]. However, the difference in molecular structure between the two kinds of materials is that polyzwitterionic materials possess highly charged groups, whereas polyhydrophilic materials have hydroxyl and ester groups, which leads to different types of surface hydration with diverse strengths. When hydrophilic materials contact with bulk water, water molecules penetrate the polymer film to form a hydrogen-bond network in the polymers (Fig. 4Ba). However, since hydrogen bonds are relatively easy to break and reform, polyhydrophilic materials usually undergo the transition from non-fouling to fouling when surface hydration changes because of the raise of temperature [204,205], the increase of hydrophobicity when copolymerized with hydrophobic monomers [206], and the increase of packing density [207,208], showing unstable non-fouling ability. For polyzwitterionic materials, the “surface-bound” waters are formed by strong electrostatically induced hydration, which has an obviously stronger hydration strength than hydrogen bonding [209]. (Fig. 4Bb).

Aside from hydration, chain flexibility also plays an important role in protein resistance, especially for long-chain polymers. This is because the steric repulsion caused by the compression of the polymer chains could resist protein adsorption [210]. (Fig. 4Bc) Along the same line, the protein-resistant ability of polyzwitterionic materials is also related to self-associations among zwitterionic moieties and the interactions between the charged groups of proteins and zwitterionic moieties [211,212]. Consequently, the homogenous mixture of balanced charge groups on their surface and the charge neutrality are critical factors in controlling the non-fouling properties of polyzwitterionic materials.

(1) Poly-zwitterionic biomaterials

Polyzwitterionic materials could be classified into two major kinds: polyampholytes carrying negatively and positively charged moieties on different monomer units, and polybetaines carrying negatively and positively charged moieties on the same monomer unit. Among them, polybetaines mainly include three types with different negatively charged groups: sulfonate-betaines (SB), carboxylate-betaines (CB), and phosphonate-betaines (PB). And, polyampholyte polymers such as mixed charge complex –SO_3_^−/−^COO^−^ and –N^+^(CH_3_)_3_ and natural amino acids (Asp^−^, Glu^−^, Arg^+^, and Lys^+^) are formed by a pair of separate monomers with two opposite charge moieties respectively. Their uniformity of charge neutrality and charge distribution are mostly achieved by 1:1 homogeneous reaction mixture of the two oppositely charged monomers before co-polymerization.

Based on the molecular mechanism of nonspecific protein adsorption resistance of zwitterionic materials and the earlier studies by Whitesides and co-workers [213], carboxybetaine (CB) and sulfobetaine (SB) materials were demonstrated to be super low fouling (<5 ng cm^−2^ adsorbed proteins [214]) in single protein solutions and undiluted blood plasma and serum [215] due to their strong hydration. Hence, many studies have been conducted to use poly-zwitterionic materials for POA prevention [216].

Ershuai et al. [18] prepared injectable zwitterion poly(carboxybetaine acrylamide) (PCBAA) solution and found that fibronectin adsorption could be entirely prevented on the zwitterionic polymer-protected injured surface of the rat abdominal wall wound *in vivo* within 24h of surgery, and the fibroblast invasion and adhesion could be remarkably reduced at day 4 after surgery. Furtherly, zwitterionic PCBAA polymer was demonstrated to completely and reliably prevent postoperative adhesion in three models (abdominal wall defect–cecum abrasion adhesion model; repeated-injury adhesion model; 70% hepatectomy adhesion model), showing better efficacy of POA prevention than that of Interceed film that Interceed film (most popular in the United States) could only slightly reduce but could not fully prevent adhesion in all these models. Meanwhile, Zhang et al. [19] crosslinked carboxybetaine acrylamide (CBAA) monomer with N, N′-bis(acryloyl) cystamine (BAC), a crosslinker containing disulfide bonds, to prepare the biodegradable zwitterionic bulk hydrogel. The zwitterionic cream gel could resist protein adsorption and fibroblast adhesion, performing outstanding postoperative adhesion prevention efficiency in both rat sidewall defect-cecum abrasion model and rat repeated-injury adhesion model. Besides, Guo et al. [217] prepared purely zwitterionic hydrogels (Z-hydrogels) with thiolated poly(sulfobetaine methacrylate-co-2-((2-hydroxyethyl)disulfanyl)ethyl methacrylate) [poly(SBMA-co-HDSMA)] as the network backbone and divinyl-functionalized sulfobetaine (BMSAB) as the zwitterionic cross-linker. The hydrogels were demonstrated to effectively suppress the formation of postoperative adhesion in the rat model of sidewall defect-cecum abrasion via reducing protein deposition and resisting fibroblast adhesion.

**Fig. 5:**
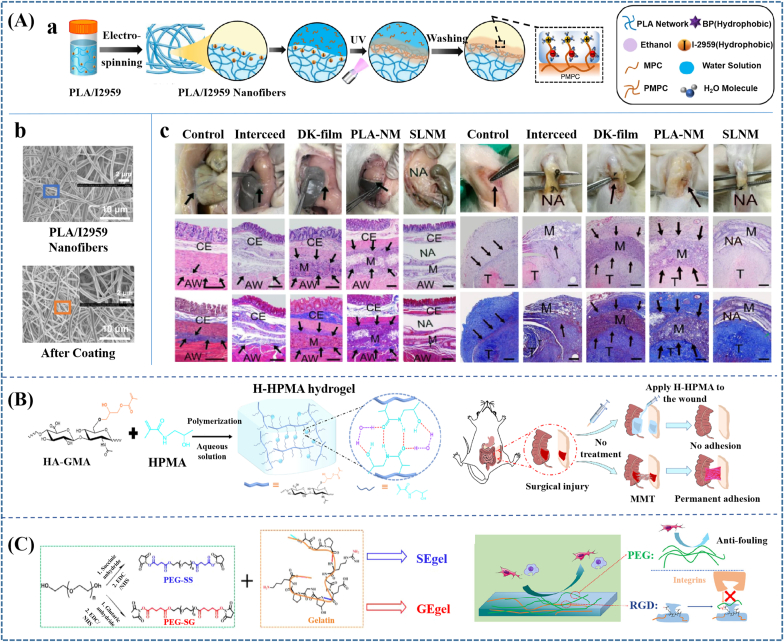
**Preventing adhesion by applying antifouling materials.** (A) Applying Poly-zwitterionic materials to achieve antiadhesive effectiveness. (a) Constructing PMPC coating on PLA membrane through subsurface-initiated approach; (b) The SEM images of PLA/I2959 nanofibers before and after coating. (c) Photos, H&E staining and Masson staining images of the harvested cecum and abdominal wall, as well as tendon on 14 d following implantation. Scale bar: 500 μm. The black arrows pointed to adhesion site; M membrane; NA no adhesion; CE cecum; AW abdominal wall; T tendon. The membranes performed antiadhesion effectiveness in both rat tendon adhesion model and abdominal adhesion model. (B) Introducing ultra-hydrophilic structure to promote antifouling capability and efficiency of postsurgical adhesions prevention; (C) Fabricating self-deactivating bio-adhesives to prevent postoperative adhesion. Reproduced with permission: (A) [219], copyright 2022, NPG; (B) [222], copyright 2022, Elsevier; (C) [200], copyright 2022, ACS.

Poly-zwitterionic materials were usually prepared as hydrogel for POA prevention, while quite a few was applied as coating to construct superlubricated surface. For example, Wang et al. [218] applied zwitterionic materials as coating of PLA membranes and achieved effective inhibition to fibroblast adhesion and remarkable decrease on tendon adhesion. In another recent study, they fabricated PLA electrospinning membranes with a poly (2-methacryloyloxyethyl phosphorylcholine) (PMPC) coating through subsurface-initiated approach [219]. (Fig. 5Aa and b) The presence of zwitterionic polymer coating endowed electrospinning membranes with hydration lubrication and antiadhesive performances. Specifically, the surface-functionalized PLA membranes performed antiadhesion effectiveness in both rat tendon adhesion model and abdominal adhesion model (Fig. 5Ac). Materials prepared through this kind of method could overcome the limitations of single hydrogels to achieve optimized performances and might be prospective for wider applications.

(2) Poly-hydrophilic biomaterials

Poly-hydrophilic materials, such as poly(N-alkyl-β-alanine), poly(ethylene glycol), polyamines, polysaccharides, and self-assembling monolayers (SAMs) have been reported great superiority in postoperative adhesion prevention due to their outstanding ability in inhibiting protein and cell adhesions [19,220]. Their antifouling ability mainly attributes to the formation of the hydration layer near the surface [221].

Yu et al. [220] introduced one extra methylene between two amides to the side chain of N-acryloyl glycinamide (NAGA) to synthesize a novel *N*-acryloyl alaninamide (NAAA) by reacting acryloyl chloride with alaninamide. Subsequently, the poly(N-acryloyl alaninamide) (PNAAA) and the supramolecular poly(N-acryloyl glycinamide) (PNAGA) hydrogel were prepared by free radical polymerization of NAAA and NAGA aqueous solution, respectively. Compared to PNAGA hydrogel, PNAAA hydrogel has weakened interpolymer H-bonds, however, increased water-polymer H-bonds. The increased water-polymer H-bonds was demonstrated to afford an excellent ability to prevent protein absorption and fibroblast adhesion. In addition, the PNAAA hydrogel revealed excellent *in vivo* antiadhesion efficacy compared to the commercial hyaluronic acid (HA) hydrogel in the rat model of sidewall defect-cecum abrasion and the recurrent adhesion model.

Introducing ultra-hydrophilic structure could also improve the antifouling capability of hydrophilic polymers, further promoting adhesion prevention ability. Guo et al. [222] introduced ultra-hydrophilic N-(2-hydroxypropyl) methacrylamide (HPMA) chains to the H-HPMA hydrogel through free-radical polymerization in an aqueous solution between the HPMA and methacrylate hyaluronic acid (HA-GMA) monomer. The introduction of the ultra-hydrophilic HPMA chains afforded a satisfying antifouling capability because of the established dense hydrated layer, furthermore, rapidly prevented the postsurgical adhesions and recurrent adhesions after adhesiolysis *in vivo* (Fig. 5B). Meanwhile, Zhao et al. [22] also incorporated highly hydrophilic HPMA with N-acryloyl glycinamide (NAGA) to fabricate injectable PNAGA-PHPMA hydrogel. The introduction of HPMA significantly reduced non-specific protein adsorption and fibroblast cell adhesion, and eventually achieved an effective anti-abdominal adhesion therapeutic effect.

(3) Self-deactivating biomaterials

It must be taken into consideration that cell adhesion mainly depends on the mutual recognition and interaction of integrin receptors and protein ligands in ECM [223]. Herein, for bioactive material, it could be speculated that if blocking the activity of the cell attachment-promoting components, such as arginine-glycine-aspartic acid (Arg-Gly-Asp) sequence (RGD), to fabricate self-deactivating biomaterials, the antifouling capability would be promoted. Wang et al. [200] mixed aqueous solutions of gelatin and poly(ethylene glycol) succinimide succinate (PEG-SS) to fabricate succinyl ester-based bioadhesive (SEgel). PEG NHS-esters in PEG-SS were demonstrated to block the RGD sequences contained in gelatin through the reaction between NHS-esters and –NH_2_ from arginine, which could prevent cell adhesion by influencing the interactions between RGD and integrins and further improve postoperative antiadhesion prevention ability (Fig. 5C). Specifically, the SEgel showed outstanding attachment resistance ability of fibrocytes and macrophages and antiadhesion properties in both the hepatic adhesion model and the cecum-sidewall adhesion model when compared to the most used glutaryl ester-based bioadhesive (SGel).

Even though there are still only some researches about applying antifouling materials for POA prevention, it could be speculated that this kind of method is prospective in preventing POA formation because of their outstanding anti-fouling ability.

### 3.3 Constructing biomaterials with ability of preventing excessive deposition of fibrin, hematoma or macrophage-fibrin clot

Even though reducing the adhesion and deposition of fibrin and cells on the material surface can inhibit the formation of adhesion tissues on the surface, if the fibrin forms in a large amount to encapsulate the material, the POA prevention could be unsuccessful. Hence, taking efforts in reducing the formation of fibrinogen and fibrin, or improving the degradation of formed fibrin to prevent excessive deposition of fibrin is required.

Fibrinogen formation is mainly related to the severity of the injury and acute inflammation, which mainly depends on the surgery technology and subsequent hemostasis and anti-inflammatory process. Meanwhile, fibrin formation is associated with the amount and activity of thrombin. And, fibrin degradation mainly depends on the fibrin system. Hence, if biomaterial can play a role in decreasing the amount and activity of thrombin, improving the amount and activity of plasmin and PA, or inhibiting the activity of PAI and PI, it will decrease fibrin deposition [224,225]. Few biomaterials have been reported to achieve these functions only by themselves. Hence, biomaterials loading related drugs or active factors might be considered first.

There are several types of drugs or active factors used in POA prevention through preventing excessive deposition of fibrin, which mainly include anticoagulation agents (*e.g.* heparin and hirudin [226]), fibrinolytic agents (*e.g.* tissue plasminogen activator (tPA) [227], pentoxifylline (PTX) [228,229], streptokinase (SK) [229,230], tranilast [231] and N-acetyl-l-cysteine [232]), PAI-1 inhibitor (*e.g.,* TM5275 [233], angiotensin type 1 (AT1) receptor antagonists [234]), anti-inflammatory agents that can restore mesothelial fibrinolytic activity (*e.g.,* simvastatin (SIM) [235,236] and 3-Hydroxy-3-Methylglutaryl Coenzyme A (HMG-CoA) reductase inhibitors [237,238]), and keratinocyte growth factor (KGF) [239] that can upregulate PMC regeneration to achieve earlier recovery of mesothelial fibrinolytic. Among them, anticoagulation agents mainly decrease the fibrin formation, yet other agents are responsible for the promotion of the fibrinolytic system. All these agents indicated obvious POA prevention efficacy. For example, Yagmurlu et al. [232] loaded SK (150,000 U) in polyhydroxybutyrate-co-hydroxyvalerate membranes and demonstrated that the membranes could prevent postsurgical adhesion formation in 90% of rats. Jackson et al. [240] combined KGF with carboxymethyl chitosan and found a synergic action that decreased postoperative pericardial adhesions. And, Tao et al. [241] prepared tPA loaded thermosensitive hydrogel and achieved an ideal anti-adhesion effect in a rat repeated-injury peritoneal adhesion model. However, most of them have been demonstrated their effects on POA prevention in animal models through oral administration, local injection or lavage, or intravenous injection (IV), only some of them have been studied in clinical and few have been applied with biomaterials.

Notably, most agents perform anti-adhesion effects depending on their doses, it is important to provide an appropriate dose. The titration studies in animal models demonstrated that the threshold dose of heparin for significant anti-adhesion effect was (without the occurrence of bleeding after two days) 7.5 × 10^−3^ U/kg/day [242]. It might be the reason why using 5000 I.U. of heparin in saline to wash the peritoneal cavity had no effect on adhesion formation [243]. However, the administration of agents, especially with high doses, is possible to have adverse effects. For example, the application of tPA and SK were reported to increase the risk of hemorrhage after surgery [244,245]. Therefore, even the biomaterials loading related agents are worthy to be studied, and the usage of these agents must be careful.

For the prevention of hematoma deposition, the improvement of the fibrinolytic system is also beneficial to its degradation. On the other hand, except for the efforts on postoperative hemostasis, biomaterials playing the role in hemostasis can also contribute to decreasing hematoma deposition. However, the avoidance of hematoma may not necessarily prevent adhesion formation, thereby biomaterials applied in POA formation usually have other functions except for hemostasis [8,246]. Whereas, these kinds of materials could be applied in the prevention of POA formation in tissues that easily occur hematoma. Besides, good effect of hemostasis can also decrease the excessive fibrin formed from coagulation process. Hence, biomaterials that can enrich coagulation components in wound sites through physical or chemical pathways such as water absorption to indirectly activate physiological hemostasis (*e.g.* gelatin, alginate), can directly activate or participate in the coagulation system (*e.g.* chitosan and its derivatives, oxycellulose, collagen), or can physically seal the vessel by strong adhesion (*e.g.* PEG) are ideal candidates [182,[247], [248], [249]].

For prevention of POA formation in serosa, inhibiting macrophage aggregation needs to be considered. However, there is still few researches preventing serosa adhesion through preventing macrophage aggregation. Wu et al. [250] prepared injectable asymmetric-adhesive hydrogel as a GATA6^+^ cavity macrophage trap, which could neutralize scavenger receptors, thereby inhibiting collagen deposition and uncontrolled recruitment of GATA6^+^ cavity macrophages. The hydrogel was demonstrated to favorably inhibit postoperative adhesion formation. This effort may give us some enlightenment on serosa adhesion prevention.

In short, applying agents is the relatively effective way to prevent excessive deposition of fibrin, hematoma or macrophage-fibrin clot. However, it is necessary to select appropriate methods according specific conditions. At present, most agents used for POA prevention have not been researched the combination with biomaterials, which need more efforts and would show prospective in POA prevention.

### 3.4 Constructing biomaterials with property of preventing excessive deposition of ECM with vascular invasion

It would be effective for POA prevention if excessive deposition of fibrin, hematoma, or macrophage-fibrin clot could be prevented thoroughly. However, in most cases, thorough prevention is difficult. In addition, cell proliferation and differentiation could be promoted under the effect of chronic inflammation. Hence, it needs to be taken into consideration to prevent excessive deposition of ECM and vascular formation.

The prevention of excessive deposition of ECM might be devoted to decreasing ECM formation or promoting ECM degradation. Among them, ECM formation is mainly attributed to the immoderate proliferation of fibroblasts and vascular formation. And, ECM degradation is related to MMPs and TIMPs. Hence, inhibiting fibroblast proliferation and vascular formation or inducing fibroblast apoptosis, increasing or activating MMPs, and decreasing TIMPs are possible to prevent excessive deposition of ECM.

There are plenty of studies about influencing fibroblast and vascular formation to prevent POA, most of which are pure agents or combination of biomaterials and agents. Celecoxib [251], 5-nitro-2 -(3-phenylpropyl amino)-benzoic acid (NPPB) [252], tioxolone [253], p-glycoprotein (P-gp) inhibitor [254], l-phenylalanine (l-Phe) [255] and low concentrations of dexamethasone [256], *etc.*, have been demonstrated to inhibit the activity or proliferation of fibroblasts and decrease collagen secretion, performing prominent anti-adhesion potential without evident side effect. However, even though most of them have been demonstrated their efficiency in preventing POA formation, only a few such as celecoxib and dexamethasone have been combined with biomaterials [248,257]. And, some of them such as NPPB still have no report in animals or humans to confirm their efficiency. Besides, 10-hydroxycamptothecine (HCPT) and mitomycin (MMC) C were reported to selectively induce cycle arrest or apoptosis of fibroblasts, thereby showing a dose-dependent adhesion prevention effectiveness [72,[258], [259], [260]]. The optimal concentration of HCPT in reducing intraarticular adhesion after knee surgery in rabbits was demonstrated to be 1.0 mg/ml when topically applicated [261]. Whereas, the side effect has been reported that peritoneal washing with MMC solutions (0.5 mg/kg and 1 mg/kg) caused animal death even though it was beneficial in decreasing the severity of adhesion band formation. And, there is still no clinical report on the application of MMC for POA formation.

Moreover, TNP-470 [262], an inhibitor of angiogenesis, sunitinib [263], an antagonist of VEGF receptor 2 (VEGFR-2) [254], and bevacizumab, an antiangiogenic recombinant mAb with the major effects on VEGF, *etc.*, have also been reported to effectively decrease the adhesion severity scores by inhibiting angiogenesis. Nevertheless, most of them have been demonstrated to have serious side effects. For example, TNP-470 was indicated to have neurotoxicity and delay wound healing [264], and bevacizumab could disrupt embryogenesis and suppress endocrine, hematopoietic, and immune systems [265].

In addition, hyaluronic acid, chitosan and its derivatives are two kinds of most used biomaterials that were reported to can inhibit the proliferation of fibroblasts and reduce the formation of collagen. The various properties of hyaluronic acid make it effective in preventing adhesion in many tissues including dura mater, pericardium, uterine, tendon, and peritoneum [[266], [267], [268], [269]]. In addition, Seprafilm® is a bioresorbable membrane composed of chemically modified sodium hyaluronate and carboxymethylcellulose, which is currently marketed as a medical product with effective anti-adhesive properties and has been used to prevent adhesions after laparotomy and cardiovascular surgery [270]. Chitosan is also widely applied in clinical. A product prepared using chitosan was demonstrated effective in preventing adhesion in 770 patients undergoing gynaecological surgery [271]. Chitosan-glucan gel (CS-DEX) and N, O-carboxymethyl chitosan (NOCC) are the two most commonly used chitosan derivatives in the field of preventing tissue adhesion [272]. Among them, NOCC was reported to perform even more obvious anti-adhesion effectiveness than hyaluronic acid [273].

Even though there are some studies about influencing fibroblast behaviors or vascular formation, few studies related to regulating MMPs or TIMPs have been conducted. In addition, only limited kinds of agents have been examined for their efficacy as a complex with biomaterials. Hence, more efforts need to be taken on constructing biomaterials with the property of preventing excessive deposition of ECM with vascular invasion.

### 3.5 Constructing biomaterials with performance of preventing abnormal remodeling of ECM

The regulation of ECM deposition or vascular formation couldn't eliminate the influence of TGF-β and mechanical signals from biomaterials. Hence, inhibiting the formation and proliferation of myofibroblast needs to be taken into consideration to prevent abnormal ECM remodeling. For anti-adhesive biomaterials, regulating chemical or mechanical clues are the two usually used methods.

**Fig. 6:**
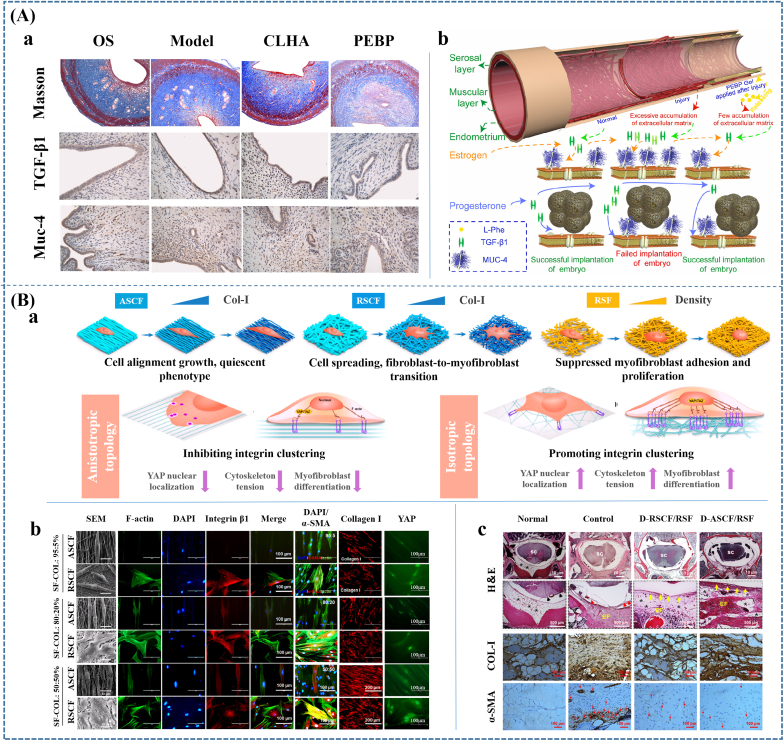
**Preventing adhesion by constructing biomaterials with performance of preventing abnormal remodeling of ECM including providing (A) chemical clues and (B) mechanical clues.** (A) Constructing biomaterials with chemical clues to inhibit fibrosis. The PEBP hydrogel group had the least fibrotic tissue and expressed least TGF-β and Muc-4 in the uterine stromal layer 10 d postsurgery compared to the sham-operated group (OS), the control uterine tube (Model) group and the cross-linked hyaluronic acid hydrogel (CLHA) group (usually applied in IUA prevention clinically) (a). Hence, it significantly decreased the degree of fibrosis might through regulating the expression and interactions of TGF-β1 and Muc-4 (b). (B) Regulating cell behavior by constructing electrospun fiber scaffolds with different microstructures and chemical cues (a). Morphology of fibroblasts and immunofluorescence staining of integrin β1, α-SMA and COL-I in fibroblasts cultured on ASCF and RSCF containing varying COL-I content (5, 20, and 50%) (b) indicated that the anisotropic scaffold could inhibit integrin clustering, reorganize cytoskeleton and reduce cell tension, inhibit YAP nuclear localization, and down-regulate α-SMA expression. Histopathological characterization of spinal dura mater and epidural tissues 8 weeks after operation (c) further showed an apparent inhibition effect of the anisotropic scaffold on COL-I and α-SMA expression, thereby to effectively prevent epidural scar adhesion. Reproduced with permission: (A) [281], copyright 2021, ACS; (B) [283], copyright 2020, AAAS.

Chemical clues usually regulated by agents loaded in biomaterials. Currently, many kinds of agents have been applied in studies of anti-adhesion prevention, which mainly include TGF inhibitors (*e.g.* TGF-β1-neutralizing mAb [144], TGF-β1 receptor kinase inhibitor EW-7197 [274], and TGF-β1 mi-RNA plasmids [275]), trametinib that inhibits the production of collagen-containing ECM and the transition to a myofibroblast phenotype in resident cells through inhibiting the activation of Erk1/2 [276], and selective small molecule activator protein (AP)-1 inhibitor T-5224 that inhibits fibrotic signaling pathways in adhesion formation via suppressing JUN signaling [1]. In addition, fucoidans [277], pirfenidone (PFD, 5-methyl-1-phenyl-2-[1H]-pyridone) [278], and tetrandrine (TET) [279] have been also demonstrated ideal anti-adhesion effects by inhibiting TGF-β1 transcription or the translation from other cells to myofibroblast or myofibroblast proliferation. Particularly, Zindel al et [126]. found that small molecule inhibitor Gefitinib could reduce the peritoneal adhesions in a mouse model by targeting epidermal growth factor receptor (EGFR) signaling, which drives the transformation from the mesothelium to myofibroblasts in post-surgical peritoneal adhesion driven by intraperitoneal microbial contamination. Cai et al. [280] encapsulated the siRNA/G5-GBA polyplexes targeted TGF-β1 in the CMCS-AGE hydrogel, which was prepared by crosslinking allyl glycidyl ether (AGE) modified carboxymethyl chitosan (CMCS-AGE) with MMP-2 substrate peptide CPLGLAGC (MMP-2 sp). The polyplexes could achieve on-demand and unidirectional delivery in response to the upregulation of MMP-2 in peritendinous tissue and inhibit fibroblasts proliferation and collagen deposition by silencing fibrosis gene TGF-β1, showing satisfactory peritendinous anti-adhesion effect. Besides, Wang et al. [281] prepared PEBP (Poly(ethylene glycol)-b-poly(l-phenylalanine))/PEG hydrogel, the hydrogel was demonstrated to suppress uterine fibrosis caused by curettage through regulating the expression and interactions of TGF-β1 and Muc-4 (Fig. 6A).

Even though there are few biomaterials that show activity in inhibiting TGF-β1 transcription or myofibroblast translation, since the process of transformation from other cells to myofibroblasts is related to mechanical signals, it could be achieved through adjusting the surface structure of biomaterials. For instance, Xu et al. [282] fabricated hierarchical micro/nanoscaled SF-PDA-COL fibrous scaffolds, the scaffolds not only acted as physical barriers to prevent adhesion but also obviously reduced α-SMA expression, thereby preventing adhesion in a biochemical way. This is mainly attributed to the special structure that nano/micro combined scaffold with good mechanical properties can resist contractions produced by fibroblasts, while self-assembly collagen on electrospun can provide substrates with soft surface properties and good compliance to prevent stimulating the transformation of fibroblasts to myofibroblast and decrease scar formation. In addition, they further made a successful attempt to regulate cell behavior by constructing electrospinning fiber scaffolds with different microstructures and chemical cues [283]. They prepared an anisotropic SF/COL-I fiber scaffold and found that it could inhibit integrin clustering, reorganize cytoskeleton and reduce cell tension, and inhibit YAP nuclear localization, down-regulating α-SMA expression and inhibiting fibroblast differentiation into the myofibroblast phenotype to prevent fibrosis (Fig. 6B). Meanwhile, the adhesion and spreading of myofibroblasts could be suppressed by increasing the density of fibers, which could significantly inhibit the contractile force of myofibroblasts. Nevertheless, the increase of COL-I concentration was demonstrated to raise the adhesion ligand density to promote higher integrin β1 activation level and induce α-SMA expression, promoting the severity of adhesion formation. These efforts might bring us inspiration to breakthrough limitations from the chemical properties of biomaterials. In other word, applying various technical means to modulate the physical properties of materials could achieve the inhibition of myofibroblast transformation from fibroblasts and their adhesion and proliferation.

In a word, the usage of chemical and mechanical signals showed obvious effectiveness in preventing POA. The existing studies can also enlighten us to integrate the two signals to design and prepared more effective anti-adhesion biomaterials.

### 3.6 Constructing materials to reduce inflammatory

Since inflammatory has a lasting impact on POA formation that it not only influences fibrinogen exudation and fibrin formation but also promotes the subsequent proliferation and differentiation of fibroblasts and the synthesis of ECM. Hence, anti-inflammatory performance has been regarded as a key factor and commonly used strategy for biomaterials applied in POA prevention. And, biomaterials and agents with anti-inflammatory effects have been widely used to prevent POA. Compared with systemic administration, biomaterials loading with anti-inflammatory components can achieve local drug delivery, promoting bioavailability and anti-inflammatory efficacy and decreasing side effects. There are main two kinds of strategies for biomaterials to achieve anti-inflammatory, which include constructing materials with negative charges on the surface and performing as a vehicle for anti-inflammatory drugs [284].

Since proinflammatory cytokines in the surrounding tissue such as IL-1β, TNF-α, IL-1α, IL- 6, IL-8, and *etc.* are positively charged, materials with negative charges on the surface can reduce their concentration levels, thereby reducing inflammation and showing anti-adhesion efficiency [285,286]. Shen et al. [287] constructed inflammation-modulating polymer scaffolds and achieved satisfactory anti-adhesion performance. The inflammation modulating function depended on the negatively charged phosphate ester crosslinked group in the in-situ phosphate crosslinked poly (vinyl alcohol) polymer (X-PVA). When the bio-scaffold was implanted in a mouse skin wound model, its effectiveness in capturing the proinflammatory cytokines was about 3 times more than that of the PROLENE mesh. Meanwhile, when it was implanted in a rat ventral hernia model with PROLENE mesh as control, the PROLENE mesh was found to trigger an extreme level of intraperitoneal adhesions formation to the mesh. Yet, no adhesion was observed in the bio-scaffold group. Meanwhile, Cui et al. [288] constructed negatively charged hydrogel by using supramolecular poly(N-acryloyl 2-glycine) (PACG), the carboxyl in the surface of which played the role in preventing post-surgical adhesion. In the gastric perforation repair experiment on rabbits, the hydrogel exhibited superior anti-adhesion efficiency compared to the surgical suture. Specifically, a white pus appeared on the suture site after the 7th day of the suture, and a mass of polyps and granulation tissues in the sewing position were formed on the 10th and 14th days with severe postoperative adhesion. However, the stomach of the hydrogel group remained smooth, and no polyps or tissue adhesions were observed on the 14th day.

Anti-inflammatory efficacy through pure biomaterials with negative charges is respectively low compared with using some anti-inflammatory drugs. Drugs that have been demonstrated to impact adhesion formation through inhibiting inflammatory are as follows. The most used drugs are dexamethasone and ibuprofen, which are FDA proved anti-inflammatory drugs and have been combined with biomaterials. Dexamethasone was used with a gelatin sponge or encapsulated within the hydrogel system, and ibuprofen was loaded in poly (l-Lactide)-polyethylene glycol (PELA) fiber membrane or mesoporous silica nanoparticles [248,[289], [290], [291]]. While most drugs have only been researched individually. Pirfenidone (PFD, 5-methyl-1-phenyl-2-[1H]-pyridone) was reported to exert anti-adhesion efficacy by regulating inflammatory pathways, such as platelet-derived growth factor, IL-1β, TNF-α, and IL-17, as well as T helper 2 and Treg cells [[292], [293], [294]]. Interleukins and interleukin inhibitors such as anti-IL-1, anti-IL-6, anti-IL-10 and anti-IL-22 have also been demonstrated to ameliorate the postoperative adhesion formation in animal models [[295], [296], [297], [298]]. In addition, R243 was indicated to reduce the rate of peritoneal adhesions by inhibiting CCL1/CCR8 interaction and further interrupting macrophage migration into the peritoneum [299,300]. And, HuoXueTongFu formula consisting of six plants (including radish seed, peach kernel, safflower, Glauber's salt, Corydalis yanhusuo, and Chinese rhubarb) was also reported to perform anti-inflammatory effect by regulating macrophage polarization [301]. Besides, some other herbal medicines with anti-inflammatory functions have also indicated their efficacy in POA prevention, which include emodin and gallic acid [302,303].

Until now, few studies have been conducted to combine constructing negatively charged surfaces and applying anti-inflammatory drugs, which is a promising direction for POA prevention.

### 3.7 Combining several strategies to construct multi-functional materials

In fact, as a result of the complicated pathogenesis of post-adhesion, simplex physical barrier or exerting influence on one particular step during adhesion formation might be difficult to meet the demand for advanced adhesion prevention and the improvement of adhesion-associated complications. Besides, some problems seriously aggravating POA formation such as bacterial infection and oxidative stress couldn't be resolved through the above strategies. Therefore, biomaterials with multi-functions, such as hemostasis, anti-inflammation, antibacterial, anti-fibrosis, and antioxidant, have been employed to achieve a synergistic effect on anti-adhesion.

**Fig. 7:**
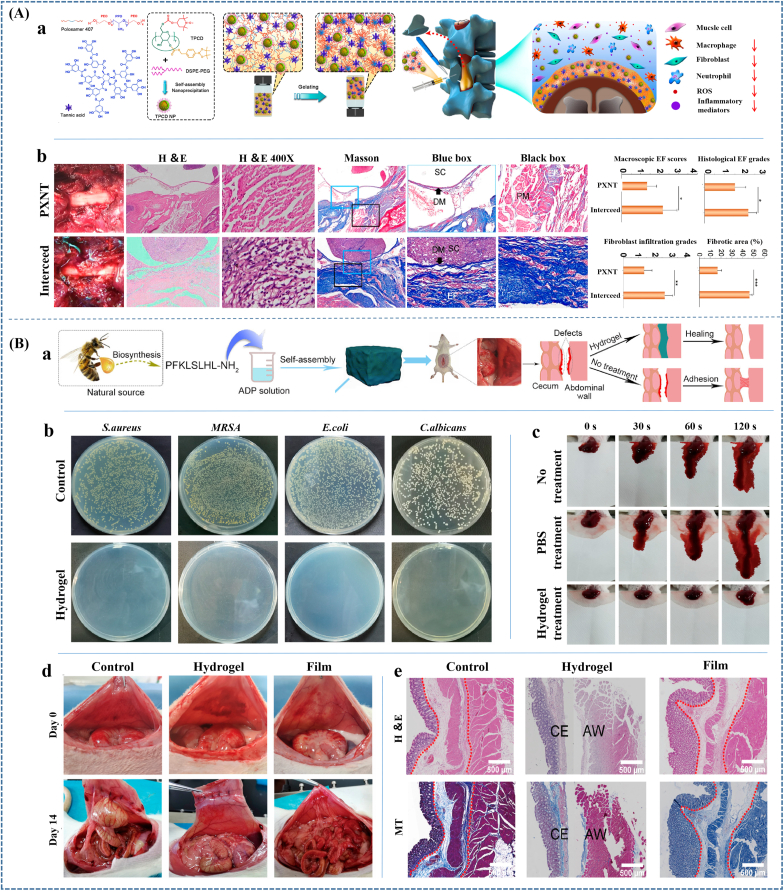
**Constructing multifunctional biomaterials to prevent POA**. (A) Engineering a multifunctional hydrogel with functions of antioxidation, anti-inflammation, and antifibrosis to prevent epidural fibrosis and adhesion after laminectomy (a). Macroscopic observation, H&E and Masson staining results, the quantified epidural fibrosis (EF) scores, fibroblast infiltration grades, and percentages of fibrotic areas after 3 weeks of treatment in rats (b) showed that PXNT hydrogel had a more beneficial anti-adhesion efficacy than that of the commercial adhesion barrier Interceed. Data are presented as mean ± SD (n = 6). *P < 0.05, **P < 0.005, ***P < 0.0005. (B) Constructing a multi-function J-1-ADP hydrogel with hemostatic and antimicrobial activity to prevent abdominal adhesion (a). The outstanding antimicrobial activity of J-1-ADP hydrogel was demonstrated from the results of colony formation of S. aureus, MRSA, E. coli, and C. albicans from the suspensions of bacteria/fungi after incubation with hydrogel for 24 h (b). And, the prominent hemostatic activity of the hydrogel was indicated in the photographs of the bleeding liver with no treated, PBS treated, and the J-1-ADP hydrogel treated groups at the indicated time interval (c). Lastly, the obvious anti-adhesion efficacy of the hydrogel could be found in the results of formation of the abdominal adhesions between the abdominal wall and cecum in the control group, J-1-ADP hydrogel treatment group, and film treatment group at day 0 and day 14 post-operation (d), and the histopathological analysis of specimens in the three groups on day 14 post-operation (e). AW: surface of the abdominal wall. CE: surface of the cecum. Reproduced with permission: (A) [7], copyright 2020, ACS; (B) [8], copyright 2022, ACS.

The most used strategy to design multifunctional materials is integrating various components with related functions [285]. For example, Shalumon et al. [304] prepared core-shell nanofibrous membranes (CSNMs) with embedded silver nanoparticles (Ag NPs) in the PEG/PCL shell and HA/ibuprofen in the core, the Ag NPs and ibuprofen among which played the role in anti-infection and anti-inflammation, respectively. The results demonstrated the outstanding performance of the multi-functional barrier in preventing peritendinous adhesion. In addition, Wang et al. [7] designed and engineered a multifunctional antiadhesion hydrogel with functions of antioxidation, anti-inflammation, and antifibrosis through integrating three functional modules, including an adhesion-enhancing compound, tannic acid (TA), a reactive oxygen species-eliminating and anti-inflammatory nanoparticle (TPCD NP), and a thermosensitive triblock copolymer, poloxamer 407 (PX) (Fig. 7Aa). This optimal formulation (PXNT) was demonstrated to effectively prevent epidural fibrosis and adhesion after laminectomy in both rats and rabbits, even showing a more beneficial efficacy than a commercially available barrier product, Interceed (Fig. 7Ab). Meanwhile, Zhao et al. [22] fabricated a supramolecular hydrogel (PNAGA-PHPMA) by copolymerization of highly hydrophilic N-(2-hydroxypropyl) methacrylamide (HPMA) and N-acryloyl glycinamide (NAGA). The hydrogel demonstrated an efficient inhibitory effect on postoperative abdominal adhesions and recurrent adhesions after adhesiolysis, which was not only as a physical barrier with outstanding antifouling capability, but also as a biochemical barrier. Specifically, it could downregulate fibrosis-related cytokines (TGF-beta 1 and fibrinogen) and pro-inflammatory cytokines (TNF-alpha and IL-6), regulate the fibrinolytic system balance (t-PA and PAI-1), and inhibit the activity of Erk1/2 and p38 kinase in the mitogen-activated protein kinase (MAPK) signaling pathway. Currently, Zeng et al. [305] combined carboxymethyl chitosan (CMCS) that exhibits favorable antibacterial effects and quercetin (Que) that exhibits favorable antibacterial effects via 2-formylphenylboronic acid (2-FPBA), a small molecule cross-linking agent. The supramolecular hybrid hydrogel performed ideal postoperative anti-adhesion ability because of its favorable antibacterial, anti-inflammatory, and antioxidant effects. Since infection and bleeding are regarded to be the incitements of adhesion formation, Zhou et al. [8] constructed a multi-function J-1-ADP hydrogel via natural antimicrobial peptide jelleine-1 (J-1) self-assembling in adenosine diphosphate (ADP, a platelet-activating factor) sodium solution (Fig. 7Ba). The hydrogel was found to have significant hemostatic activity, good antimicrobial activity against the bacteria and fungi tested, and can be used to prevent tissue infection, showing a significant antiadhesion effect in a rat side wall defect–cecum abrasion model (Fig. 7Bb-e). Li et al. [306] also prepared antibacterial, hemostasis, adhesive, self-healing polysaccharides-based composite hydrogel, which was composed of N, O-carboxymethyl chitosan (N,O-CS) and oxidized dextran (ODA). Results demonstrated that the multifunctional hydrogel could effectively prevent post-operative peritoneal adhesion without side effects. It could be found that preparing multifunctional biomaterials have been a prospective trend for POA prevention.

Apart from the integration of biomaterials, it is worth noting to apply of some agents with multi-functions. As discussed above, chitosan and its derivatives perform activities of hemostatic, antibacterial and anti-fibrosis, and can be considered as a key component of anti-adhesion material preparation [306]. In addition, ghrelin (a gastric peptide) was reported to show anti-fibrotic and anti-inflammatory activities and has been demonstrated to reduce the formation of post-operative abdominal adhesions via down-regulating the pro-inflammatory gene and protein expression (*e.g.,* TGF-β3 and TGFβ-R2) [307,308]. YC-1(3-[5′-Hydroxymethyl-2′-furyl]-1-benzyl-indazole), a small molecule HIF inhibitor, was demonstrated to effectively prevent postsurgical peritoneal adhesions by attenuating pro-inflammatory activation of macrophages, impairing recruitment and activation of peritoneal fibroblasts, mitigating epithelial-mesenchymal-transition (EMT), as well as enhancing fibrinolysis and impairing angiogenesis [309].

Notably, some agents derived from naturally sourced herbs perform multi bioactivities with light side effects and can also be used in anti-adhesion prevention. Ligustrazine nanoparticles (from the root of Rhizoma chuanxiong) were demonstrated to inhibit POA formation through their antioxidant and anti-inflammatory activities [310]. Tanshinone IIA was also reported to reduce the adhesion scores in rats because of the reduction on levels of PAI, COX-2 messenger ribonucleic, collagen I and TGF-β1, as well as the enhancement of the tPA concentration [295,311]. Breviscapine (a crude extract of several flavonoids of Erigeron breviscapus) can inhibit the POA formation through the modulation of IL-6, IL-18, and TNF-α in serum and PAI-1, TGF-β1, as well as connective tissue growth factor in the peritoneal fluid [312]. Meanwhile, bromelain (crude extract of pineapple) was reported to suppress the adhesion formation in rats by decreasing inflammation, neovascularization, and fibrosis [313]. Bletilla striata (aqueous extraction) was demonstrated to show activities of anti-inflammation, hemostasis and anti-fibrosis. These multi functions attribute to its efficacy in POA prevention. Also, rosmarinus officinalis (aerial parts) have been demonstrated to increase the level of the anti-oxidative factor (glutathione) and decrease oxidative factors (nitricoxide and malondialdehyde), inflammatory cytokines (*e.g.*, IL-β1, TNF-α, TGF-β1, and IL-6), and angiogenesis biomarker (VEGF) [314]. Particularly, curcumin, a natural polyphenol molecule derived from the rhizome of turmeric has been demonstrated anti-inflammatory, antibacterial and antioxidant activities. Experiments have found that its anti-inflammatory effect can even be comparable to some steroid hormones [[315], [316], [317], [318], [319], [320], [321]]. Moreover, it can achieve anti-fibrotic effect through six mechanisms, including (1) Inhibiting the proliferation of fibroblasts and inducing apoptosis; (2) Reducing ECM deposition; (3) Regulating the activities of matrix metalloproteinases and their inhibitors; (4) Blocking TGF-β signal transduction pathway; (5) Resisting oxidation; (6) Improving the function of cystic fibrosis transmembrane transduction regulators [140]. These mentioned functions provide curcumin tremendous potential in POA prevention. However, even though curcumin as medicine has been proven effective in POA prevention in many studies, there are still few studies about integrating biomaterials to prevent POA formation [322,323]. Consequently, further studies need to be implemented.

Most of these herbs have been only studied as medicine, thereby combining them with other biomaterials still requires much efforts.

## 4 Summary and prospect

Even though the reported researches about antiadhesive biomaterials have demonstrated their efficacy on POA inhibition to a certain extent, thoroughly preventing POA formation is still challenging. To optimize the POA prevention efficacy of materials, it is necessary to understand the mechanisms of POA occurrence and development so that to apply medicine according to indications. Therefore, we summarized and analyzed the process of the occurrence and development of POA and proposed seven strategies for POA prevention though using biomaterials based on the mechanisms. Meanwhile, the relevant practices were summarized depending on the corresponding strategies.

It is no doubt that the more in-depth level of research on the mechanism of POA formation, the better to precisely guide the design and preparation of materials. Currently, non-specific drugs were reported to inhibit adhesion formation with few effectiveness and even cause some adverse effects. However, the discussed mechanisms are macroscopic universal mechanisms among various tissues. And, the mechanism researches in the level of cells, molecules, or signaling pathways are still indistinct and insufficient. Even though fibroblasts and myofibroblasts might be regarded as the main cells impacting the formation of “vascular adhesion”, “adhesive adhesion” and “scarred adhesion” in various tissues, cells in the original stage of “membranous adhesion” under different pathological conditions performed specificity. Whereas, except for the key role of cavity macrophages and mesothelial cell in the formation of peritoneal adhesions, few studies revealed the specific cells in various stages [54]. Hence, in-depth mechanisms of POA formation in different level for diverse tissues still need to be studied.

The study of mechanism relies on advanced technology and equipment. The *in vitro* assay developed for peritonea adhesion pathology research by Adrian al et. could simulate adhesiogenesis between human organ surfaces in microscopic detail [64]. It successfully circumvented the difficulties of visualizing germinal events in live animals, and would be prospectively applied in other tissues. Meanwhile, the new *in vivo* imaging system (intravital imaging of mouse peritoneal cavities) applied in the study of cavity macrophages might inspire other studies [54].

Up to now, various materials have been applied for POA prevention. For ideal effect, it is better to fabricate antiadhesive materials with the foundational functions of both inhibiting cells crossing and reducing the adhesion of fibrin and cells. Methods and techniques for constructing physical barriers are relatively well established and widely applied, while the research and application of reducing the adhesion of fibrin and cells are still insufficient. Although related theories have been provided, there were limited studies about prevent POA through constructing superhydrophobic surface or applying antifouling materials. Hence, it would be promising to optimize the composition and surface structure of antiadhesive materials through using poly-zwitterionic biomaterials and poly-hydrophilic biomaterials, fabricating self-deactivating biomaterials and superhydrophobic surface. Technologies to construct specific surface also need to pay more attention [324].

In addition, the existing practices indicated that applying pure biomaterials is difficult to achieve ideal POA prevention effectiveness. However, recent studies about cell therapy and alternating capacitive electric fields to regulate tissue repair might provide us with some new perspectives [[325], [326], [327]]. Moreover, drugs or small-molecule inhibitors, such as Tocilizumab, an anti-human IL-6Rα monoclonal antibody [296], and medicines from natural sources with fewer side effects need to be taken into consideration. However, the extent of side effects is usually related to the dose of drugs, and only limited drugs have been researched [261,328]. Hence, more efforts need to be taken to obtain the safe and effective doses of drugs used in specific tissues for POA prevention. Moreover, the practical dose of drugs loaded in biomaterials depends on the released drugs. However, most agents for POA prevention have been only studied alone without integration with biomaterials. Besides, imposing obstacles at different stages during adhesion formation through various agents to achieve a more effective POA prevention effect requires precise regulation of the agents. Hence, medicine loading and controlled release need to be regarded as the key points. The study of Cai et al. on drug release in an intelligently responsive way is worthy of reference. [11] And, applying materials (*e.g.* carbon-based nanomaterials [329,330]) easy to load drugs to develop novel antiadhesive biomaterials and developing intelligent drug delivery systems that adapt to the dynamics of complex drug release are promising directions [331,332].

Meanwhile, since POA is the consequence of abnormal tissue repair, it is difficult for anti-adhesive materials to achieve normal tissue repair. On the contrary, they can usually inhibit tissue repair. Hence, multifunctional materials that can simultaneously promote normal regeneration and prevent adhesion, and even treat particular disease (*e.g.* tumor [333]) are the inevitable development trend of anti-adhesive materials, which needs the innovation in material system. Constructing double-layer or multilayer materials with excellent structural stability is the most used strategy. Among them, Janus porous hydrogel [175,288], hydrogel-electrospinning system [177], electrospinning-electrospinning system [334], electrospinning-cast film system [335], electrospinning-sheet system [336,337], sponge-hydrogel system [338], aerogel-electrospinning system [339] and layer-by-layer surface coating [340] show superiority and need wider application and research. In addition, the surface coating technology such as “hydrogel skin” [219,341] exhibited priority.

Notably, POA prevention in different tissues has diverse requirements in the mechanical and biochemical properties of anti-adhesive biomaterials. For example, solid barriers or barriers with high swelling rates are not appropriate for the delicate spinal cord. It is because a slight compression could result in severe neurological signs. And, barriers applied in tendon need a smooth surface to prevent friction. Materials used in peritoneal adhesion prevention may possess high tensile strength to resist cyclic mechanical forces. Hence, the design of anti-adhesive materials must suit the local conditions to meet the specific requirements.

Overall, with the unresolved reality of the frequent occurrence of POA in soft tissues and their complications, new anti-adhesive materials must be developed to provide more effective anti-adhesive effects from the perspective of inhibiting the occurrence and development of adhesions.

## Ethics approval and consent to participate

I confirm that I have obtained all consents required by applicable law for the publication of any personal details or images of patients, research subjects or other individuals that are used in the materials submitted to KeAi. I have retained a written copy of all such consents and I agree to provide KeAi with copies of the consents or evidence that such consents have been obtained if requested by KeAi.

## Declaration of competing interest

There are no conflicts of interest to declare.
